# Health Behaviours, Socioeconomic Status, and Mortality: Further Analyses of the British Whitehall II and the French GAZEL Prospective Cohorts

**DOI:** 10.1371/journal.pmed.1000419

**Published:** 2011-02-22

**Authors:** Silvia Stringhini, Aline Dugravot, Martin Shipley, Marcel Goldberg, Marie Zins, Mika Kivimäki, Michael Marmot, Séverine Sabia, Archana Singh-Manoux

**Affiliations:** 1 INSERM U1018, Centre for Research in Epidemiology and Population Health, Hôpital Paul Brousse, Villejuif, France; 2 Department of Epidemiology and Public Health, University College London, London, United Kingdom; 3 Centre de Gérontologie, Hôpital Sainte Périne, Assistance Publique-Hôpitaux de Paris, Paris, France; The University of Queensland, Australia

## Abstract

**Background:**

Differences in morbidity and mortality between socioeconomic groups constitute one of the most consistent findings of epidemiologic research. However, research on social inequalities in health has yet to provide a comprehensive understanding of the mechanisms underlying this association. In recent analysis, we showed health behaviours, assessed longitudinally over the follow-up, to explain a major proportion of the association of socioeconomic status (SES) with mortality in the British Whitehall II study. However, whether health behaviours are equally important mediators of the SES-mortality association in different cultural settings remains unknown. In the present paper, we examine this issue in Whitehall II and another prospective European cohort, the French GAZEL study.

**Methods and Findings:**

We included 9,771 participants from the Whitehall II study and 17,760 from the GAZEL study. Over the follow-up (mean 19.5 y in Whitehall II and 16.5 y in GAZEL), health behaviours (smoking, alcohol consumption, diet, and physical activity), were assessed longitudinally. Occupation (in the main analysis), education, and income (supplementary analysis) were the markers of SES. The socioeconomic gradient in smoking was greater (*p*<0.001) in Whitehall II (odds ratio [OR]  = 3.68, 95% confidence interval [CI] 3.11–4.36) than in GAZEL (OR  = 1.33, 95% CI 1.18–1.49); this was also true for unhealthy diet (OR  = 7.42, 95% CI 5.19–10.60 in Whitehall II and OR  = 1.31, 95% CI 1.15–1.49 in GAZEL, *p*<0.001). Socioeconomic differences in mortality were similar in the two cohorts, a hazard ratio of 1.62 (95% CI 1.28–2.05) in Whitehall II and 1.94 in GAZEL (95% CI 1.58–2.39) for lowest versus highest occupational position. Health behaviours attenuated the association of SES with mortality by 75% (95% CI 44%–149%) in Whitehall II but only by 19% (95% CI 13%–29%) in GAZEL. Analysis using education and income yielded similar results.

**Conclusions:**

Health behaviours were strong predictors of mortality in both cohorts but their association with SES was remarkably different. Thus, health behaviours are likely to be major contributors of socioeconomic differences in health only in contexts with a marked social characterisation of health behaviours.

*Please see later in the article for the Editors' Summary*

## Abstract 2

Further analysis of data from two prospective cohorts reveals differences in the extent to which health behaviors attenuate associations between socioeconomic position and mortality outcomes.

## Editors' Summary

**Background:**

The influence of the socioeconomic environment on the health of individuals and populations is well known, giving rise to the so-called social determinants of health. The social determinants of health are the conditions in which people are born, grow, live, work, and age, including the health system. These circumstances are shaped by the distribution of money, power, and resources at global, national, and local levels, which are themselves influenced by policy choices. The social determinants of health are mostly responsible for health inequities—the unfair and avoidable differences in health status seen within and between countries. In addition, health-damaging behaviors are often strongly socially patterned. For example, material constraints, lack of knowledge, and limited opportunities to follow health promoting messages often act as barriers that prevent those from lower socioeconomic groups to adopt a healthy lifestyle. Yet the extent to which health behaviors explain social inequalities in health remains unclear and can range from 12% to 72% according to some studies.

**Why Was This Study Done?:**

In a recently published paper using data from the British Whitehall II cohort, the researchers showed that longitudinal assessment of health behaviors accounted for socioeconomic differences in mortality better than a single baseline assessment as used in most previous studies. (The Whitehall II study started in 1985 to examine the socioeconomic gradient in health among 10,308 London-based civil servants [6,895 men and 3,413 women] aged 35–55).

However, it is not clear whether health behaviors are equally important mediators of the socioeconomic-health association in different cultural settings. In this study, the researchers examine this issue by comparing their recent findings of the Whitehall II study with another European cohort, the French GAZEL study. (The GAZEL study started in 1989 among employees of the French national gas and electricity company totaling 20,625 employees [15,011 men and 5,614 women], aged 35–50.) The Whitehall II study and the GAZEL study have comparable designs in the way both assess socioeconomic status, health behaviors, and mortality and have a similar age range and follow-up period.

**What Did the Researchers Do and Find?:**

The researchers included 9,771 participants from the Whitehall II study and 17,760 from the GAZEL study—mean follow up for Whitehall II was 19.5 years and for GAZEL was 16.5 years. The researchers used occupation as the main marker of socioeconomic status, and education and income as supplementary markers of socioeconomic status. Apart from a few exceptions, the researchers analyzed each cohort separately and used statistical techniques to calculate: the mortality rates per 1000 person-years for each socioeconomic group; the age- and sex-adjusted prevalence rates of smoking, heavy alcohol consumption, unhealthy diet, and physical inactivity, at the first and the last follow-up of the study for each socioeconomic group; and the differences in health behaviors prevalence between lowest and highest occupational position. Then the researchers used a statistical model to deduce the contribution of all health behaviors.

The researchers found that the socioeconomic gradient in smoking, unhealthy diet, and physical inactivity was greater in Whitehall II than in GAZEL. Socioeconomic differences in mortality were similar in the two cohorts, a hazard ratio of 1.62 in Whitehall II and 1.94 in GAZEL for lowest versus highest occupational position. Health behaviors weakened the association between socioeconomic status and mortality by 75% in Whitehall II but only by 19% in GAZEL. The supplementary analysis the researchers conducted using education and income as socioeconomic markers gave similar results.

**What Do These Findings Mean?:**

These results suggest that the social patterning of unhealthy behaviors differs between countries. Although in both cohorts socioeconomic status and health behaviors were strong predictors of mortality, major differences in the social patterning of unhealthy behaviors in the two cohorts meant that the causal chains leading from socioeconomic status to health behaviors to mortality were different. Therefore it may be that health behaviors are likely to only be major contributors of socioeconomic differences in health in contexts with a marked social characterization of those behaviors. In order to identify the common and unique determinants of social inequalities in health in different populations, there needs to be further comparative research on the relative importance of different pathways linking socioeconomic status to health.

**Additional Information:**

Please access these websites via the online version of this summary at http://dx.doi.org/10.1371/journal.pmed.1000419.

- WHO provides information on social determinants of health
- University College London provides information on the Whitehall study
- The GAZEL study is available in an online open access format

## Introduction

Differences in morbidity and mortality between socioeconomic groups constitute one of the most consistent findings of epidemiologic research [1]. The first comprehensive investigation of the reasons behind social inequalities, the Black Report, identified four possible explanations: artefactual, natural or social selection, materialist/structural, and cultural/behavioural [2]. Much subsequent research, although recognising the existence of socioeconomic differences, has yet to provide a complete understanding of the mechanisms behind the association between markers of socioeconomic status (SES) and health [3],[4]. A better understanding of these mechanisms is essential in order to identify targets for intervention aimed at reducing social inequalities in health.

Lifestyle and health-related behaviours are major determinants of the population distribution of health and disease [5]–[13]. Health damaging behaviours are often strongly socially patterned; material constraints, lack of knowledge, and limited opportunities to take up health promoting messages may act as barriers for lower socioeconomic groups to adopt a healthy lifestyle [2],[14]–[17]. However, the extent to which health behaviours explain social inequalities in health remains unclear [18]–[23], as their estimated contribution ranges from 12% to 72% [24]–[29] in various studies. In a recently published paper using data from the British Whitehall II cohort [29], we showed that longitudinal assessment of health behaviours accounted for socioeconomic differences in mortality better than a single baseline assessment as is the case in most previous studies [24]–[28].

Longitudinal measurement appears to be important, but whether health behaviours are equally important mediators of the SES-health association in different cultural settings remains unknown and is the object of investigation in this paper. Our previous research was based on the British Whitehall II study [29]; here we examine the generalisability of our findings by also including data from another European cohort, the French GAZEL study. In order to understand the causal chain leading to social inequalities in mortality we extend previous analysis by examining the social patterning of health behaviours over the follow-up and the association between health behaviours and mortality in the two cohorts, the French GAZEL and the British Whitehall II cohorts. These studies have comparable design with regards to assessment of SES, health behaviours, and mortality and have a similar age range and follow-up period.

## Methods

### Ethics Statement

The University College London ethics committee approved the Whitehall II study. The GAZEL study received ethical approval from the French national ethics committee (Commission nationale de l'informatique et des libertés [CNIL]).

### Study Populations

The Whitehall II study was established in 1985 to examine the socioeconomic gradient in health among 10,308 London-based civil servants (6,895 men and 3,413 women) aged 35–55 [30]. Baseline examination (phase 1) took place during 1985–1988, and involved a clinical examination and a self-administered questionnaire containing sections on demographic characteristics, health, lifestyle factors, work characteristics, social support, and life events.

The GAZEL study was established in 1989 among employees of the French national gas and electricity company, Electricité de France-Gaz de France (EDF-GDF) [31]. At baseline (1989), 20,625 employees (15,011 men and 5,614 women), aged 35–50, gave consent to participate. The study design consists of an annual questionnaire used to collect data on health, lifestyle, individual, familial, social and occupational factors, and life events.

### Socioeconomic Status

Occupation, education, and income are the standard markers used to characterise SES. In the main results of the paper we use occupational position as a marker of SES as it not only represents the occupational hierarchy but is also related to income, education, level of responsibility in the job, and retirement benefits. Furthermore, misclassification, measurement error, or missing data are less likely on this measure due to linkage with employers' records. However, as SES is a multifaceted concept we provide analyses using education (Tables S1–S4) and income (Tables S5–S8) as measures of SES.

In both cohorts we used occupational position from the baseline of the studies. In Whitehall II this was the British civil service employment grade; representing high (administrative), intermediate (professional or executive), and low (clerical or support) grades. In GAZEL, we used the employer's (EDF-GDF) categorisation representing high (managers), intermediate (skilled workers), and low (unskilled workers) occupational position. It must be noted that the Whitehall II cohort comprises only white-collar workers while the GAZEL cohort also includes blue-collar workers. Although the main analysis in the paper uses all available data, sensitivity analysis including only the white-collar workers in the GAZEL cohort is presented in Tables S9 to S12.

### Health Behaviours

In the Whitehall II study, data on health behaviours were drawn from phases 1 (1985–1988), 3 (1991–1993), 5 (1997–1999), and 7 (2002–2004) of the study. Data missing at one phase were replaced with data from one phase immediately prior or subsequent to that phase.

In the GAZEL study, data on health behaviours were drawn from three time windows: 1990–1995, 1996–2001, and 2002–2007. Information on smoking status and alcohol consumption was available yearly and these data were drawn primarily from a single year in each time window (1992, 1998, and 2004) with data from the previous or successive years used to replace missing values. Information on diet and physical activity was available at least once in each time window. Data missing at one of the time windows were replaced using information from the closest time window.

Smoking status was categorized as current and noncurrent smokers, the latter category including both never smokers and ex-smokers. Alcohol consumption in both cohorts was assessed via questions on the number of alcoholic drinks consumed in the previous week. This was converted to number of alcohol units (1 unit corresponds to 8 g of alcohol) consumed per week [32]. Participants were categorized as “abstainers” (0 unit/wk), “moderate” (1–21 units/wk for men, 1–14 for women), and “heavy drinkers” (>21 units/wk for men, >14 for women) [33]. Dietary patterns were assessed, in Whitehall II, via questions on the frequency of fruit and vegetable consumption and participant's diet was classified as “unhealthy” for those eating fruit and vegetables less than three times a month; “healthy” for those eating fruit and vegetables at least once a day; or “moderately healthy” for dietary pattern in between these two extremes. In GAZEL, they were assessed via questions on the frequency of consumption of fruit and green vegetables (first time window) and on the frequency of consumption of fruit and cooked and raw vegetables (second and third time windows). Participant's diet was classified as “healthy” if they reported eating fruit and vegetables “almost every day”; “unhealthy” if they reported eating fruit and vegetables “seldom or never”; or “moderately healthy” if their dietary pattern was in between these two extremes. Physical activity was assessed, in Whitehall II, at phases 1 and 3 using questions on the frequency and duration of participation in mildly energetic, moderately energetic, and vigorous physical activity. At phases 5 and 7, the questionnaire was modified to include 20 items on frequency and duration of participation in different physical activities used to compute hours per week of each intensity level. These data were used to classify participants as “active” (>2.5 h/wk of moderate physical activity or >1 h/wk of vigorous physical activity), “inactive” (<1 h/wk of moderate physical activity and <1 h/wk of vigorous physical activity), or “moderately active” (if not active or inactive). In GAZEL, physical activity was assessed with a question on participation in sports activities with respondents classified as “physically active” if they practised sports regularly (at least once a week), “inactive” if they did not report participation in any sports activities, and “moderately active” if they reported participating in sports activities only occasionally.

### Mortality Follow-up

Whitehall II study: 10,297 (99.9%) respondents were successfully traced and have been followed for mortality through the national mortality register kept by the National Health Services Central Registry, using the National Health Service identification number assigned to each British citizen. Participants were followed-up for mortality from their entry in the study until 30th April 2009; a mean of 19.5 y.

GAZEL study: vital status data on all participants are obtained annually from EDF-GDF itself as it pays out retirement benefits. The follow-up in our analysis starts in 1992 as data on weekly consumption of alcohol are only available from that year. Mortality follow-up was available until 30th September 2009, a mean of 16.5 y.

### Statistical Analysis

With the few exceptions mentioned below, all analyses were performed separately in the two cohorts. First, we calculated the mortality rates per 1,000 person-years for each socioeconomic group, standardized for age (4–5-y age groups) and sex with the direct method. Then, we used least squares regression to calculate age- and sex-adjusted prevalence rates of smoking, heavy alcohol consumption, unhealthy diet, and physical inactivity, at the first and the last follow-up of the study, for each socioeconomic group, and differences in health behaviours prevalence between lowest and highest SES.

The association of SES with each health behaviour at the first and last measurement over the follow-up period was examined using age- and sex-adjusted log-binomial and logistic regression. Cox proportional regression analysis was used to estimate hazard ratios (HRs) and their 95% confidence intervals (CIs) for the association between each health behaviour, used as time-dependent variable, and mortality. We also estimated HRs and their 95% CIs for the association between SES and mortality. A first model included adjustment for age at baseline and sex (model 1). Subsequently, smoking status, alcohol consumption, dietary patterns, and physical activity assessed longitudinally through the follow-up were entered individually and then simultaneously into the model. The same procedure was repeated using additive models, and rate differences between lowest and highest occupational position were calculated before and after adjustment for health behaviours. The contribution of each health behaviour in explaining the association between SES and mortality was determined by the percent reduction in the coefficient for SES (for both additive models and Cox regressions) after inclusion of the health behaviour in question to model 1, using the formula “100× (β_Model 1_ − β_Model 1 _+ health behaviour(s))/(β_Model 1_)”. The contribution of all health behaviours was deduced from the model adjusted for all health behaviours in a similar manner. In order to add precision to the percent attenuation we calculated a 95% CI around it using a bias corrected accelerated (BCa) bootstrap method with 2000 resamplings [34].

Participants with complete data, after imputation, on all health behaviours at all intervals preceding death or the end of follow-up were censored at their date of death or at end of follow-up. The remaining participants were censored at the last date at which they had complete data on all health behaviours for all preceding intervals. The proportional hazard assumptions for Cox regression models were tested using Schoenfeld residuals and found not to be violated (all *p*-values ≥0.05).

As tests did not suggest departures from a linear trend (all *p*-values ≥0.05), in both cohorts we used the measure of SES as a continuous three-level variable. The odds (or hazard) ratio associated with a unit change in SES was squared to yield the odds (or hazard) ratio for the lowest versus the highest socioeconomic group (a two-unit change) under the assumption of linearity of association between SES and behaviours (or between SES and mortality).

In order to test whether the associations between SES and health behaviours, between health behaviours and mortality, and between SES and mortality differed in the two cohorts, an interaction term between SES and cohort was fitted in the different regression models described above including both cohorts.

The main analysis was performed using the statistical software STATA 10 (StataCorp LP). BCa confidence intervals were calculated using the statistical software SAS 9 and the %BOOT and %BOOTCI macros (http://support.sas.com/kb/24/982.html).

## Results

In the Whitehall II study, a total of 537 participants, corresponding to 5% of the total population (4% men and 8% women), were excluded from the analysis because they had missing data on health behaviours at baseline (ten for smoking, 94 for alcohol consumption, 33 for fruit and vegetables consumption, and 416 for physical activity, categories not mutually exclusive) or had not been followed up for mortality (11 participants). The analysis was based on the remaining 9,771 participants (68% male and 32% female). Those excluded tended to have a lower occupational position at baseline (42% versus 22% in the lowest occupational group, *p<*0.001) and had a higher mortality rate (4.7 per 1,000 person-years versus 3.6 in the included sample). There were no age differences between the included and excluded men (44.0 versus 44.2 y, *p = *0.6); excluded women were older (47.0 versus 45.1 y, *p<*0.001). Nonincluded participants had in general worse health behaviours than those included in the analysis. In the GAZEL study, a total of 2,865 participants, corresponding to 14% of the total population (13% men and 16% women), were excluded from the analysis because they had missing data on occupational position (25 participants) or on health behaviours at baseline (132 for smoking, 23 for alcohol consumption, 1,861 for fruit and vegetables consumption, and 2,091 for physical activity) or died before the start of the follow-up in 1992 (91 participants), all categories not mutually exclusive. The analysis was based on the remaining 17,760 participants (76% male and 26% female). Those excluded tended to have a lower occupational position (28% versus 16% in the lowest group, *p<*0.001) at baseline, and had a higher mortality rate (6.6 per 1,000 person-years versus 3.1 in the included sample). There were no age differences between the included and nonincluded sample although the latter had in general worse health behaviours.

Table 1 shows the sample characteristics in the two studies. The distribution of participants across the occupational groups was similar in the two cohorts (21.6% of participants were in the lowest socioeconomic group in Whitehall II and 16.3% in GAZEL). A clear social gradient in mortality across the socioeconomic groups was evident in both studies. The overall mortality rate per 1,000 person-years was slightly greater in Whitehall II (3.6 versus 3.1 per 1,000 person-years in Whitehall II and GAZEL, respectively).

**Table 1:** Sample characteristics of the British Whitehall II and the French GAZEL cohort studies.

Study	Occupational Position			Overall
	High	Intermediate	Low	
**Whitehall II**				
N (%)	2,914 (29.8)	4,744 (48.6)	2,113 (21.6)	9,771
Deaths (Rate^a^)	197 (3.1)	322 (3.8)	174 (5.2)	693 (3.6)
Mean age (SD)	45.0 (5.8)	43.3 (6.0)	46.0 (6.0)	44.4 (6.1)
**GAZEL**				
N (%)	4,497 (25.3)	10,365 (58.4)	2,898 (16.3)	17,760
Deaths (Rate^a^)	210 (2.6)	518 (3.1)	180 (4.6)	908 (3.1)
Mean age (SD)	44.2 (3.2)	43.3 (3.5)	42.3 (3.7)	43.4 (3.5)

a Age- and sex-adjusted mortality rate per 1,000 person-years.

SD, standard deviation.

**Figure 1:**
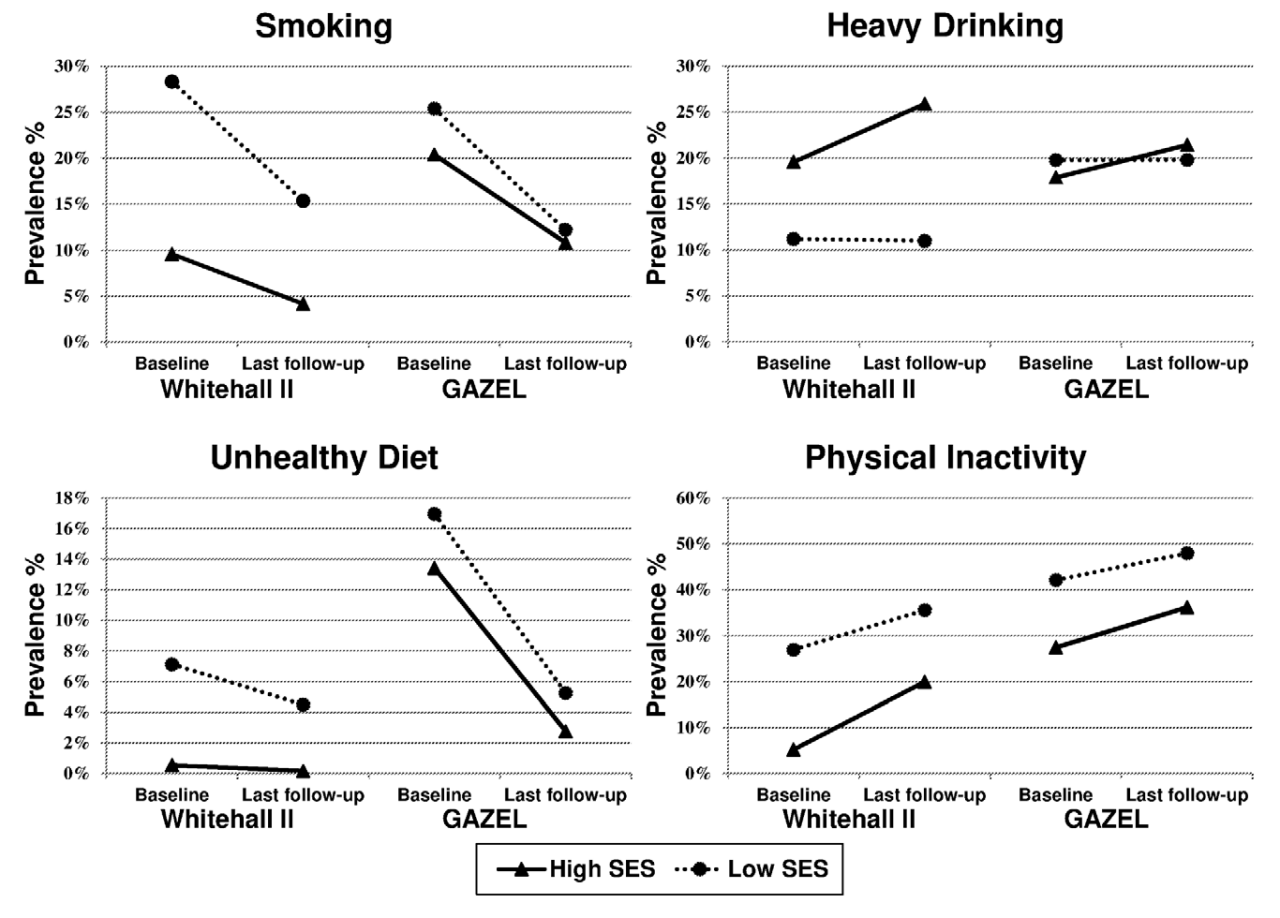
Age- and sex-adjusted prevalence of unhealthy behaviours at baseline and at last follow-up as a function of occupational position (high SES and low SES) in the British Whitehall II and the French GAZEL cohorts.

Age- and sex-adjusted prevalence rates of unhealthy behaviours at baseline and at last follow-up as a function of occupational position are shown in Figure 1. The prevalence of smoking and of unhealthy diet declined in both cohorts, particularly in GAZEL, in both the highest and the lowest occupational categories. Over the same time period, the prevalence of physical inactivity increased in both cohorts.

Results for the association of occupational position with health behaviours assessed at the first (phase 1 for Whitehall II and first time window for GAZEL) and last follow-up (phase 7 for Whitehall II and last time window for GAZEL) are presented in Table 2. In terms of absolute differences, in Whitehall II, apart from heavy drinking, unhealthy behaviours at baseline were more prevalent in the lowest comparing to the highest occupational group; there was a 19% (95% CI 16%–21%) absolute difference between these groups for smoking, 7% (95% CI 5%–8%) for following an unhealthy diet, and 22% (95% CI 20%–24%) for being physically inactive. In GAZEL, the prevalence of unhealthy behaviours was also greater in the lowest occupational group but absolute differences were smaller than in the Whitehall II study. The difference in prevalence between the highest and the lowest occupational groups was 5% (95% CI 3%–7%) for smoking, 4% (95% CI 2%–5%) for following an unhealthy diet, and 15% (95% CI 12%–17%) for being physically inactive. At the end of follow-up, great inequalities persisted in Whitehall II in smoking, following an unhealthy diet and being physically inactive. In contrast, there were only small differences between socioeconomic groups in all health behaviours apart from physical inactivity in GAZEL.

**Table 2:** Association of occupational position with health behaviours in the British Whitehall II (*n = *9,771 at first and *n = *7,166 at last follow-up) and the French GAZEL (*n = *17,760 at first and *n = *15,377 at last follow-up) cohort studies.

Follow-up	Whitehall II				GAZEL			*p* d
	Prevalence Δ (95% CI)^a^		RR (95% CI)^b^	OR (95% CI)^c^	Prevalence Δ (95% CI)^a^	RR (95% CI)^b^	OR (95% CI)^c^	
**First follow-up**								
Smoking	19% (16%–21%)		2.88 (2.51–3.30)	3.68 (3.11–4.36)	5% (3%–7%)	1.24 (1.14–1.35)	1.33 (1.18–1.49)	<0.001
Heavy drinking	−8% (−11% to −6%)		0.58 (0.50–0.67)	0.50 (0.42–0.60)	2% (0%–4%)	1.11 (1.01–1.22)	1.14 (1.01–1.28)	<0.001
Unhealthy diet	7% (5%–8%)		6.71 (4.81–9.36)	7.42 (5.19–10.60)	4% (2%–5%)	1.25 (1.12–1.40)	1.31 (1.15–1.49)	<0.001
Physically inactive	22% (20%–24%)		4.40 (3.74–5.17)	6.07 (5.00–7.36)	15% (12%–17%)	1.52 (1.43–1.62)	1.95 (1.76–2.16)	<0.001
**Last follow-up**								
Smoking	11% (9%–13%)		3.67 (2.88–4.69)	4.17 (3.17–5.47)	1% (0%–3%)	1.13 (0.98–1.30)	1.14 (0.97–1.34)	<0.001
Heavy drinking	−15% (−18% to −12%)		0.46 (0.39–0.53)	0.36 (0.30–0.44)	−2% (−4% to 0%)	0.93 (0.85–1.03)	0.91 (0.80–1.03)	<0.001
Unhealthy diet	4% (3%–5%)		9.41 (5.46–16.21)	9.99 (5.66–17.63)	2% (1%–3%)	1.85 (1.44–2.38)	1.91 (1.47–2.49)	<0.001
Physically inactive	16% (12%–19%)		1.84 (1.63–2.09)	2.27 (1.92–2.70)	12% (9%–14%)	1.32 (1.25–1.41)	1.63 (1.47–1.81)	<0.001

a Difference in health behaviour prevalence between lowest and highest occupational position adjusted for age and sex.

b Risk ratio (RR) for lowest versus highest occupational position adjusted for age and sex.

c OR for lowest versus highest occupational position adjusted for age and sex.

d *p* for interaction between health behaviour and cohort in logistic regression.

Δ, difference.

In terms of relative differences, both risk ratios and odds ratios suggest that participants in the lowest occupational group compared to those in the highest were more likely to be smokers, follow an unhealthy diet, and be physically inactive in both cohorts at the first assessment of behaviours (Table 2). In the GAZEL but not the Whitehall II cohort, they were also more likely to be heavy drinkers. The lower half of Table 2 shows the association between occupation and health behaviours at the last follow-up for participants with complete data on all health behaviours at all follow-ups. In Whitehall II, the social patterning remained similar to that observed at the start of the follow-up, apart from physical inactivity where the association with occupational position was somewhat attenuated (OR  = 2.27, 95% CI 1.92–2.70). In GAZEL, there remained a social gradient only for unhealthy diet and physical inactivity. The association between occupational position and health behaviours was different in the two cohorts at first and at last follow-up (all *p*<0.001 for interaction between occupation and cohort).

Table 3 presents results for the association between health behaviours, used as time-dependent variables, and mortality. The relative hazards for smoking (HR  = 2.38, 95% CI 1.99–2.85 in Whitehall II and HR  = 2.10, 95% CI 1.81–2.43 in GAZEL), heavy drinking (HR  = 1.25, 95% CI 1.02–1.52 in Whitehall II and HR  = 1.16, 95% CI 0.99–1.36 in GAZEL), an unhealthy diet (HR 2.14, 95% CI 1.49–3.07 in Whitehall II and HR 2.04, 95% CI 1.61–2.60 in GAZEL), and being physically inactive (HR  = 1.60, 95% CI 1.34–1.90 in Whitehall II and HR  = 1.68, 95% CI 1.44–1.96 in GAZEL) were similar in both cohorts (all *p*>0.35 for interaction with health behaviours). Table S13 presents absolute rate differences in mortality for each health behaviour.

**Table 3:** The association between health behaviours and all-cause mortality in the British Whitehall II (*n = *9,771, deaths  = 693) and the French GAZEL (*n = *17,760, deaths  = 908) cohort studies.

Health Behaviours	Whitehall II	GAZEL	*p* d
	HR (95% CI)^a^	HR (95% CI)^a^	
**Smoking**			
Nonsmokers	1.00	1.00	
Current smokers	2.38 (1.99–2.85)	2.10 (1.81–2.43)	0.36
**Drinking**			
Abstainers	1.56 (1.30–1.87)	1.88 (1.58–2.24)	
Moderate drinkers	1.00	1.00	
Heavy drinkers	1.25 (1.02–1.52)	1.16 (0.99–1.36)	0.70
**Diet**			
Healthy	1.00	1.00	
Moderately healthy	1.41 (1.20–1.65)	1.17 (0.99–1.38)	
Unhealthy	2.14 (1.49–3.07)	2.04 (1.61–2.60)	0.49
**Physical activity**			
Active	1.00	1.00	
Moderately active	1.05 (0.86–1.30)	1.23 (1.02–1.48)	
Inactive	1.60 (1.34–1.90)	1.68 (1.44–1.96)	0.45

a Model adjusted for age at baseline and sex.

b *p* for interaction between health behaviour and cohort.

Results for the role of health behaviours in explaining the associations between occupational position and mortality are presented in Table 4. The HR for lowest versus highest occupational position was 1.62 (95% CI 1.28–2.05) in Whitehall II and 1.94 (95% CI 1.58–2.39) in GAZEL in the model adjusted for age and sex (*p* for interaction for cohort differences  = 0.92). Smoking reduced the HR by 32% (95% CI 20%–62%) in the Whitehall II study and by 4% (95% CI 2%–8%) in the GAZEL study. Diet and physical activity lowered the HR respectively by 25% (95% CI 12%–55%) and 21% (95% CI 11%–43%) in the Whitehall II study and by 4% (95% CI 2%–8%) and 8% (95% CI 4%–12%) in the GAZEL study. Overall, health behaviours explained 75% (95% CI 44%–149%) of the association between occupational position and all-cause mortality in the Whitehall II study and 19% (95% CI 13%–29%) in the GAZEL study. Table S14 shows absolute mortality differences between lowest and highest occupational groups in the two cohorts, and the percent attenuation of these differences after inclusion of health behaviours in the models. Although the absolute differences were not significant at conventional levels, the contribution of health behaviours to social inequalities in mortality using absolute differences was similar to that using relative differences (the percent attenuation in Whitehall II in the fully adjusted model was 78% for absolute differences and 75% for relative differences; in GAZEL it was 19% for both absolute and relative differences).

**Table 4:** Role of health behaviours used as time-dependent covariates in explaining the association between occupational position and all-cause mortality in the British Whitehall II (*n = *9 771, deaths  = 693) and the French GAZEL (*n = *17 760, deaths  = 908) cohort studies.

Model	Whitehall II		GAZEL	
	HR (95% CI)	Percent Δ (95% CI)a,b	HR (95% CI)	Percent Δ (95% CI)a,b
Model 1^c^	1.62 (1.28–2.05)		1.94 (1.58–2.39)	
Model 1+ smoking	1.39 (1.09–1.75)	32 (20–62)	1.89 (1.54–2.32)	4 (2–8)
Model 1+ alcohol	1.52 (1.19–1.93)	14 (3–37)	1.85 (1.51–2.28)	7 (4–11)
Model 1+ diet	1.44 (1.13–1.83)	25 (3–37)	1.89 (1.54–2.33)	4 (2–8)
Model 1+ physical activity	1.47 (1.16–1.86)	21 (11–43)	1.84 (1.50–2.27)	8 (4–12)
Fully adjusted model^d^	1.13 (0.88–1.44)	75 (44–149)	1.71 (1.39–2.10)	19 (13–29)

a Percent attenuation in log HR  = 100× (β_Model 1_ − β_Model 1_+ health behaviour(s))/(β_Model 1_), where β =  log(HR).

b Bias corrected accelerated bootstrap 95% CI.

c HR for lowest versus highest occupational position adjusted for age at baseline and sex.

d HR for lowest versus highest occupational position adjusted for age at baseline, sex, and all health behaviours.

### Analysis Using Other Markers of SES

Occupation is the most comprehensive and complete (in terms of missing data) marker of SES in both cohorts. However, in order to ensure that the results held across other markers of SES, we repeated the analysis using education and income and these yielded largely similar results to those presented in the paper. Table 5 summarizes these additional results. Briefly, health behaviours explained 56% of the education-mortality association in the Whitehall II study compared to 17% in the GAZEL study (for full results using education as a marker of SES, see Tables S1–S4). The results using income as a marker of SES were similar; health behaviours explained 56% of the social gradient in mortality in the Whitehall II study and 23% in the GAZEL study (for full results using income as a marker of SES, see Tables S5–S8). These results, although based on smaller numbers because of missing data, largely replicated those obtained using occupation, leading us to conclude that the results were robust to the way SES was measured. Table 5 also summarizes results for analyses conducted on the subsample of GAZEL workers who had white-collar occupations. These results were very similar to those observed on the whole cohort (for full results on analysis based only on white-collar workers in GAZEL, see Tables S9–S12).

**Table 5:** The contribution of health behaviours in explaining the social gradient in mortality by occupational position, education, and income in the British Whitehall II and the French GAZEL study.

Study	HR (95% CI)^a^	Smoking^b^	Drinking^b^	Diet^b^	Physical activity^b^	All behaviours^b^
**Whitehall II**						
Occupational position (*n = *9,771)	1.62 (1.28–2.05)	32%	14%	25%	21%	75%
Education (*n = *9,754)	1.43 (1.15–1.79)	31%	7%	21%	8%	56%
Income (*n = *9,671)	1.90 (1.49–2.41)	26%	10%	16%	19%	56%
**GAZEL**						
Occupational position, whole cohort (*n = *17,760)	1.94 (1.58–2.39)	4%	7%	4%	8%	19%
Occupational position, white-collar workers only (*n = *8,079)	2.26 (1.63–3.13)	3%	7%	4%	5%	17%
Education (*n = *17,449)	1.56 (1.26–1.91)	3%	4%	7%	7%	17%
Income (*n = *17,131)	2.05 (1.60–2.63)	4%	7%	5%	10%	23%

In Whitehall II, education categorized as university, secondary, and primary education was collected at phase 5 (1997–1999) and was available on 6,776 participants. The remaining participants, *n = *2,978, were imputed using multiple imputation. Income was not available at study baseline (1985–1988). We thus use a proxy measure composed of measures of car ownership and type of accommodation. The highest category represents participants owning a car and their house, the lowest represents participants not owning a car and living in rented accommodation. The intermediate category represents other combinations of car ownership and type of accommodation. In the GAZEL study education and income were collected at study baseline (1989). Education was categorized as university, secondary, and primary education. For income, the following three categories (based on quintiles of income, converted in Euro from French Francs) were used in the analysis: <1,600€, 1,600€–3,800€, and ≥3,800€.

a HR for lowest versus highest occupational position adjusted for age at baseline and sex.

b Percent attenuation in log HR  = 100× (β_Model 1_ − β_Model 1_+ health behaviour(s))/(β_Model 1_), where β =  log(HR).

### Analysis of the Impact of Missing Data

We examined the social gradient in mortality in those excluded from the analysis owing to missing data on health behaviours at baseline and found it to be similar to that reported in Table 4 (*p* for interaction for occupational position and inclusion status  = 0.35 in the Whitehall II study and 0.58 in GAZEL).

In the GAZEL study, a greater proportion of data on health behaviours was missing, 14% compared to 5% in the Whitehall II study. As this is a potential source of bias we repeated the analysis using only two of the health behaviours examined, smoking and alcohol consumption, which were available on 99% of the sample in both studies; 10,195 participants in Whitehall and 20,454 participants in GAZEL. First, we examined whether the role of these two behaviours in explaining occupational differences in mortality in the larger sample for which they were available was similar to that reported in the main analysis. These results show that in the Whitehall II study smoking contributes to 28% and alcohol consumption to 15% of the social gradient in mortality (compared to 32% and 14% in the main analysis, Table 4). In the GAZEL study, smoking explained 6% of the social gradient in mortality compared to 4% in the main analysis (Table 4), the contribution of alcohol did not differ.

In a second set of analysis, we examined the association of occupational position with smoking and alcohol consumption in participants who were not included in the main analysis. In Whitehall II, the association between occupational position and smoking in those not included (OR  = 3.78) was similar to that in the included sample (OR of 3.68) except for “heavy alcohol consumption” where it was more pronounced among those not included in the analysis (an OR of 0.19 versus an OR of 0.50). In the GAZEL study the occupational gradients in smoking (an OR of 1.19 versus an OR of 1.33) and heavy drinking (an OR of 0.84 versus an OR of 1.14) were slightly weaker in the nonincluded sample.

Finally, we used “inverse probability weighting” to correct the estimates for nonresponse [35]. Here the first step involved fitting a model to predict the probability of inclusion for each participant using covariates, drawn from the baseline of the studies, available on the whole cohort. The analysis was then rerun using weighted regression models employing weights equal to the inverse of the predicted probabilities obtained in the first step. In both cohorts, results using inverse probability weighting for missing data were similar to the results reported in the paper (see Tables S15 and S16 for results on the associations between occupational position and health behaviours and between occupational position and mortality).

### Comparison with Our Previously Published Paper

Our previous paper, based only on the Whitehall II study [29], aimed to compare the role of heath behaviours when assessed once to that assessed longitudinally in explaining SES differences in mortality. The main objective of the present paper is the comparison between Whitehall II and the GAZEL studies. Results on the Whitehall II study reported in the present paper differ slightly from those previously reported [29], as we harmonized some measures in the present analysis to allow better comparison with the GAZEL cohort. In particular, the diet variable was modified to only include data on fruit and vegetable consumption. Our previous paper also included data on the type of bread and milk consumed, but as these were not available in the GAZEL study, the measure of diet was simplified for the present analysis. The measure of smoking was coded as current or not current smoker in the present paper (not as current/ex/never smoker as in our previous publication) to obtain an identical measurement with the GAZEL data. The harmonization procedure led to less missing data at baseline in Whitehall II (537 individuals compared to 707) compared to the previously published paper.

### Additional Sensitivity Analyses

As the study design of the two cohorts was somewhat different, we conducted further analyses to test the robustness of our results. First, the Whitehall II study has a longer follow-up period (19.5 y versus 16.5 y in GAZEL), providing the participants a longer period to change their health behaviours. In order to assess possible bias we repeated the Whitehall II analysis reported in Table 4 using a similar follow-up as that in GAZEL by starting the follow-up in Whitehall II at phase 3 (1991–1993). These results showed health behaviours to explain an even greater part of the socioeconomic gradient in mortality (91%). In the second set of sensitivity analysis, we repeated the analyses on participants with complete data on all health behaviours at all follow-ups; these results did not much differ from those reported in Table 4 (for example, the percent attenuation for the fully adjusted model was 60% in Whitehall II and 17% in GAZEL). The third set of sensitivity analyses relates to social mobility in GAZEL. EDF-GDF, the employer of GAZEL's participants, had a policy of seniority-based promotion. Thus, the highest socioeconomic category is likely to include a fair proportion of individuals whose behaviours might reflect their first occupational position. In order to assess this possibility, analyses were repeated using occupational position at entry into EDF-GDF, usually when individuals are in their 20s, instead of that at the start of the GAZEL study. These results did not differ from those reported in Table 4 (for example, smoking explained 4% of the socioeconomic gradient). Finally, analyses were also repeated using pack years of smoking instead of smoking status and here again results did not differ from those presented in this study.

## Discussion

Our principal objective in these analyses was to examine whether the finding that health behaviours explained a large proportion of the association between SES and mortality in a British cohort was generalisable to other contexts. The comparison cohort in the present analysis was the French GAZEL cohort. Our hypothesis that health behaviours explain most of the social inequalities in mortality was not replicated. These results need to be interpreted in light of the fact that the associations between socioeconomic factors and mortality and that between health behaviours and mortality were similar in both cohorts. Thus, in both cohorts, SES and health behaviours were strong predictors of mortality. However, the causal chain leading from SES to health behaviours to mortality was not played out in a similar manner in the two contexts because of major differences in the social patterning of unhealthy behaviours. Indeed, relative and absolute inequalities across socioeconomic groups in smoking, following an unhealthy diet, and being physically inactive were greater in the British Whitehall II than in the French GAZEL study. As a consequence, health behaviours were less important mediators of the SES-mortality association in the GAZEL study.

It is important to consider the implications of these findings for policies aimed at improving population health, which at first appear fairly straightforward. Our results show that health behaviours are important determinants of mortality in both the French and the British contexts. In fact, the similar associations with mortality in the two cohorts suggest that population health could be improved by targeting health behaviours irrespective of the cultural context. However, population-wide interventions to improve health behaviours may result in greater uptake of the message in socially advantaged groups, potentially increasing social inequalities in health [36]. As population health is the sum of the health in various subgroups, including socioeconomic groups, an increase in social inequalities in health may in the long term impact population health. Our results suggest that policies that target unhealthy behaviours in the socially disadvantaged groups are likely to lead to decreases in social inequalities in health in the Whitehall II cohort but not in the GAZEL cohort, as health behaviours are not major mediators of the SES-health association in GAZEL. The large differences between the two cohorts in the role of health behaviours in explaining the SES-mortality association further suggest that policies specifically aimed at reducing social inequalities in health need to be based on a better understanding of the mechanisms that link socioeconomic factors to health. In particular, cultural differences and context-specific characteristics need to be taken into account as they are likely to play a role in the social distribution of unhealthy behaviours within a given population.

We have previously shown [29] that study design matters; repeated measurements of health behaviours over the study period explained a significantly greater part of the association between SES and mortality compared to a baseline-only assessment of behaviours. In the present study we used longitudinal measures of behaviours in both cohorts. Differences in the social patterning of unhealthy behaviours in the two cohorts can be related to cultural differences between the two countries. In Northern European regions a strong socioeconomic gradient in health behaviours has frequently been observed [37]–[39]. In Southern European regions smoking, eating, and drinking habits seem to be more related to cultural norms than to socioeconomic factors [40]–[42], and weak or inexistent socioeconomic gradients are frequently reported [42]–[47]. Cultural norms and traditions related to the adherence to the Mediterranean diet and moderate alcohol consumption may in part explain these north-south differences [7],[42],[43],[48]. There are also north-south differences within countries; in the GAZEL cohort the prevalence of obesity, hypercholesterolemia, and hypertension is lower among participants living in Southern France [49].

Another plausible explanation for differences in the social distribution of unhealthy behaviours might be differences in the epidemiological transition from “diseases of affluence” to the diseases of the poor [50]–[52]. Smoking and eating fatty or refined foods were once more common among the better-off [50],[51],[53]; the reversal in the social gradient in smoking, drinking, and unhealthy eating may not have happened simultaneously in the two countries. Results on relative and absolute inequalities in smoking support the hypothesis that at study inception in the 1980s, France and Britain were, and still remain, at different stages of the smoking epidemic [41]; Britain is at a stage where the prevalence of smoking has decreased substantially, particularly in the higher socioeconomic groups and France is at a stage where the prevalence of smoking is higher with no association between SES and smoking. Studies conducted in Southern European regions either show smoking to be more common in the higher socioeconomic groups or report a weak socioeconomic gradient [46],[54]–[56]. If differences in the social distribution of unhealthy behaviours between GAZEL and Whitehall II are due to the fact that the respective countries are at a different stage of the epidemiological transition, it is possible that social inequalities in behaviours in France will appear in future generations. Furthermore, country-specific factors such as price of commodities, bans on smoking, welfare policies or health promoting policies, taxes, or other factors likely to have an effect on behaviours may in turn influence their social characterisation.

In this study, we show that, not surprisingly, health behaviours contribute little to socioeconomic inequalities in mortality when their social patterning is weak. Other factors are likely to play an important role in the SES-mortality association in both cohorts. Material deprivation or financial insecurity, work stress and work environment, psychosocial factors such as job control or social support, or differential access to health care may be other possible mechanisms through which SES influences health [57]–[70]. Moreover, GAZEL includes both blue and white-collar workers; it is therefore possible that physical occupational hazards and working conditions contribute more to socioeconomic differences in health in this cohort [67],[71]. However, restricting the analysis in GAZEL to only the white-collar workers did not substantially change the results (see Tables S9–S12).

### Strengths and Weaknesses

This study has several strengths. First, we used repeated measures of health behaviours over the follow-up to account for changes that may have occurred during the study period. Second, we provide a CI for the effect of health behaviours on the socioeconomic gradient in mortality, allowing us to add a degree of precision around the estimate of the attenuations. Third, we were able to compare results from two cohorts using approximately the same measures for SES and health behaviours and an identical analytical strategy. Moreover, although the main analysis uses occupational position as a marker of SES, the results using education and income are largely similar. Finally, as a previous study using the data from the original Whitehall study [72] highlighted the importance of absolute measures of social inequalities, we used both the absolute and the relative differences approach; similarity in results from both these models adds further validity to our findings.

There are also important limitations to this study. First, two types of error need to be taken into account in interpreting our results. One, confounding by variables that are unknown or not included in the analysis may contribute to an overestimation of the role of health behaviours in the SES-mortality association [73]. If the magnitude of this overestimation is different in the two cohorts it would bias the comparison of the percent attenuation attributed to health behaviours. Two, greater measurement error in the health behaviours in one cohort compared to the other, say GAZEL compared to Whitehall II, would result in an underestimation of the percent attenuation attributed to health behaviours in GAZEL. Another consequence of greater measurement error would be that the association between health behaviours and mortality would also be underestimated. As the association between health behaviours and mortality was similar in the two cohorts, measurement error alone cannot explain the results. It seems unlikely that the biases described above would have been of a magnitude that could explain the observed differences in the percent explained by health behaviours in the two cohorts in the present study. Third, we did not examine other possible mediators of the SES-health association and cannot comment on the relative importance of health behaviours in relation to other mediators. Four, comparative research requires some compromises in the quality of the measures. One example is the comparability of eating patterns in the two populations. We chose to use a simple measure, fruit and vegetable consumption, in order to allow comparability of diet between the cohorts.

The final limitation relates to the fact that both cohorts are occupational cohorts based on individuals with stable jobs; the study design excludes those who are unemployed or have temporary jobs. In both cohorts the proportion of participants holding a university degree is similar to that of the respective general populations but participants with only primary education are underrepresented [74]. The mean yearly income per household in GAZEL was similar to the national figure in 1989 [75], while in Whitehall II household total yearly income (collected in 1997–1999) was greater than the average equivalised disposable household income in the United Kingdom. As both cohorts represent a relative advantaged fraction of the population, socioeconomic differences in morbidity and mortality are likely to be underestimated in this study [76]. However, the crucial issue for the present analysis is not whether the socioeconomic distribution in the two cohorts is comparable to the general population but whether the social patterning of health behaviours (strong SES-health behaviour association in the Whitehall II study and a weak association in the GAZEL study) is generalisable to the British and French populations. On this question, there is evidence from other studies showing a strong social patterning in health behaviours in Northern European regions including Britain but not in the Southern European countries such as France [43],[77]–[81].

### Conclusions

Health behaviours were strong predictors of mortality in both cohorts but their association with SES was remarkably different. Thus, they are likely to be major contributors of socioeconomic differences in health only in contexts with a marked social characterisation of health behaviours. Further comparative research on the relative importance of different pathways linking SES to health is needed to identify the common and unique determinants of social inequalities in health in different populations.

## Supporting Information

Table S1 **Education.** Sample characteristics of the British Whitehall II and the French GAZEL cohort studies.
(0.03 MB DOC)

Table S2 **Education.** The association of education with health behaviours in the British Whitehall II (*n  = *9,754 at first and *n = *7,163 at last follow-up) and the French GAZEL (*n = *17,449 at first and *n = *15,130 at last follow-up) cohort studies.
(0.03 MB DOC)

Table S3 **Education.** The association between health behaviours and all-cause mortality in the British Whitehall II (*n = *9,754, deaths  = 691) and the French GAZEL (*n = *17,449, deaths  = 881) cohort studies.
(0.04 MB DOC)

Table S4 **Education.** The role of health behaviours used as time-dependent covariates in explaining the association between education and all-cause mortality in the British Whitehall II (*n = *9,754, deaths  = 691) and the French GAZEL (*n = *17,449, deaths  = 881) cohort studies.
(0.03 MB DOC)

Table S5 **Income.** Sample characteristics of the British Whitehall II and the French GAZEL cohort studies.
(0.03 MB DOC)

Table S6 **Income.** The association of income with health behaviours in the British Whitehall II (*n = *9,671 at first and *n = *7,099 at last follow-up) and the French GAZEL (*n = *17,131 at first and *n = *14,859 at last follow-up) cohort studies.
(0.03 MB DOC)

Table S7 **Income.** The association between health behaviours and all-cause mortality in the British Whitehall II (*n = *9,671, deaths  = 689) and the French GAZEL (*n = *17,131, deaths  = 870) cohort studies.
(0.04 MB DOC)

Table S8 **Income.** The role of health behaviours used as time-dependent covariates in explaining the association between income and all-cause mortality in the British Whitehall II (*n = *9,671, deaths  = 689) and the French GAZEL (*n = *17,131, deaths  = 870) cohort studies.
(0.03 MB DOC)

Table S9 **GAZEL white-collar workers.** Sample characteristics of the British Whitehall II and the French GAZEL cohort studies.
(0.03 MB DOC)

Table S10 **GAZEL white-collar workers.** The association of occupational position with health behaviours in the British Whitehall II cohort (*n = *9,771 at first and *n = *7,166 at last follow-up) and in white-collar workers of the French GAZEL cohort (*n = *8,079 at first and *n = *6,902 at last follow-up).
(0.03 MB DOC)

Table S11 **GAZEL white-collar workers.** The association between health behaviours and all-cause mortality in the British Whitehall II cohort (*n = *9,771, deaths  = 693) and in white-collar workers of the French GAZEL cohort (*n = *8,079, deaths  = 350).
(0.04 MB DOC)

Table S12 **GAZEL white-collar workers.** The role of health behaviours used as time-dependent covariates in explaining the association between occupational position and all-cause mortality in the British Whitehall II cohort (*n = *9,771, deaths  = 693) and in white-collar workers of the French GAZEL cohort (*n = *8,079, deaths  = 350).
(0.03 MB DOC)

Table S13 **Absolute probabilities and absolute differences in probabilities.** The association between health behaviours and all-cause mortality in the British Whitehall II (*n = *9,771, deaths  = 693) and the French GAZEL (*n = *17,760, deaths  = 908) cohort studies.
(0.05 MB DOC)

Table S14 **Absolute probabilities and absolute differences in probabilities.** The role of health behaviours used as time-dependent covariates in explaining the association between occupational position and all-cause mortality in the British Whitehall II (*n = *9,771, deaths  = 693) and the French GAZEL (*n = *17,760, deaths  = 908) cohort studies.
(0.04 MB DOC)

Table S15 **Inverse probability weighted.** Association of occupational position with health behaviours in the British Whitehall II (*n = *9,771 at first and *n = *7,166 at last follow-up) and the French GAZEL (*n = *17,760 at first and *n = *15,377 at last follow-up) cohort studies.
(0.03 MB DOC)

Table S16 **Inverse probability weighted.** Role of health behaviours used as time-dependent covariates in explaining the association between occupational position and all-cause mortality in the British Whitehall II (*n = *9,771, deaths = 693) and the French GAZEL (*n = *17,760, deaths  = 908) cohort studies.
(0.03 MB DOC)

Text S1 **Results from supplementary analyses.** Tables S1–S16 in a single file for reader's convenience.
(0.29 MB DOC)

## Abbreviations

CI: confidence interval
EDF-GDF: Electricité de France-Gaz de France
HR: hazard ratio
OR: odds ratio
SES: socioeconomic status
