# Supplementary material for: Health Behaviours, Socioeconomic Status, and Mortality: Further Analyses of the British Whitehall II and the French GAZEL Prospective Cohorts
Source: PLoS Med. 2011 Feb 22;8(2):e1000419. doi: 10.1371/journal.pmed.1000419 (PMC3043001; doi:10.1371/journal.pmed.1000419)
Supplement: Table S1 — Education. Sample characteristics of the British Whitehall II and the French GAZEL cohort studies. (0.03 MB DOC) [file pmed.1000419.s001.doc]

Table S1 EDUCATION. Sample characteristics of the British Whitehall II and the French GAZEL cohort studies.

|  | **Education** | | | **Overall** |
| --- | --- | --- | --- | --- |
|  | Tertiary | Secondary | Primary |  |
| **WHITEHALL II** |  |  |  |  |
| N (%) | 2430 (24.9) | 5232 (53.6) | 2092 (21.5) | 9 754 |
| Deaths (Rate^a^) | 105 (2.3) | 385 (4.5) | 201 (3.2) | 691 (3.6) |
| Mean age (SD) | 42.6 (5.8) | 43.3 (5.6) | 49.0 (5.1) | 44.4 (6.1) |
| **GAZEL** |  |  |  |  |
| N (%) | 3448 (19.8) | 10299 (59.0) | 3702 (21.2) | 17449 |
| Deaths (Rate^a^) | 145 (2.5) | 513 (3.1) | 223 (3.8) | 881 (3.1) |
| Mean age (SD) | 43.4 (3.5) | 43.2 (3.5) | 43.8 (3.5) | 43.4 (3.5) |

SD=Standard Deviation

^a^ Age- and sex-adjusted mortality rate per 1000 person-years

Note: In Whitehall II, education categorized as university, secondary and primary education was collected at Phase 5 (1997/99) and was available on 6776 participants. The remaining participants N=2978 were imputed using multiple imputation.
