# Supplementary material for: Health Behaviours, Socioeconomic Status, and Mortality: Further Analyses of the British Whitehall II and the French GAZEL Prospective Cohorts
Source: PLoS Med. 2011 Feb 22;8(2):e1000419. doi: 10.1371/journal.pmed.1000419 (PMC3043001; doi:10.1371/journal.pmed.1000419)
Supplement: Table S2 — Education. The association of education with health behaviours in the British Whitehall II (n = 9,754 at first and n = 7,163 at last follow-up) and the French GAZEL (n = 17,449 at first and n = 15,130 at last follow-up) cohort studies. (0.03 MB DOC) [file pmed.1000419.s002.doc]

Table S1 EDUCATION. Association of education with health behaviours in the British Whitehall II (N=9754 at first and N=7163 at last follow-up) and the French GAZEL (N=17449 at first and N=15130 at last follow-up) cohort studies.

|  | **WHITEHALL II** | **GAZEL** | P^b^ |
| --- | --- | --- | --- |
|  | **OR^a^ (95%CI)** | **OR^a^ (95%CI)** |  |
| **FIRST FOLLOW-UP** | | | |
| Smoking | 3.85 (3.22, 4.61) | 1.21 (1.08, 1.35) | *<0.001* |
| Heavy drinking | 0.80 (0.66, 0.95) | 1.15 (1.02, 1.30) | *<0.001* |
| Unhealthy diet | 3.80 (2.62, 5.51) | 1.57 (1.38, 1.80) | *<0.001* |
| Physically inactive | 2.73 (2.62, 5.51) | 1.62 (1.47, 1.80) | *<0.001* |
| **LAST FOLLOW-UP** | | | |
| Smoking | 2.86 (2.21, 3.70) | 0.99 (0.85, 1.16) | *<0.001* |
| Heavy drinking | 0.62 (0.52, 0.74) | 1.06 (0.93, 1.20) | *<0.001* |
| Unhealthy diet | 3.00 (1.79, 5.06) | 1.75 (1.35, 2.28) | *=0.02* |
| Physically inactive | 1.22 (1.04, 1.43) | 1.44 (1.30, 1.60) | *=0.02* |

OR=Odds Ratio; CI=Confidence Interval

^a^ Odds ratio for lowest versus highest education, adjusted for age and sex

^b^ P for interaction between health behaviour and cohort
