# Supplementary material for: Health Behaviours, Socioeconomic Status, and Mortality: Further Analyses of the British Whitehall II and the French GAZEL Prospective Cohorts
Source: PLoS Med. 2011 Feb 22;8(2):e1000419. doi: 10.1371/journal.pmed.1000419 (PMC3043001; doi:10.1371/journal.pmed.1000419)
Supplement: Table S3 — Education. The association between health behaviours and all-cause mortality in the British Whitehall II (n = 9,754, deaths = 691) and the French GAZEL (n = 17,449, deaths = 881) cohort studies. (0.04 MB DOC) [file pmed.1000419.s003.doc]

Table S1 EDUCATION. The association between health behaviours and all-cause mortality in the British Whitehall II (N=9754, Deaths=691) and the French GAZEL (N=17449, Deaths=881) cohort studies.

|  | **WHITEHALL II** | **GAZEL** | P^b^ |
| --- | --- | --- | --- |
|  | **HR** ^a^ **(95% CI)** | **HR** ^a^ **(95% CI)** |  |
| **Smoking** |  |  |  |
| Non smokers | 1.00 | 1.00 |  |
| Current smokers | 2.38 (1.99, 2.85) | 2.12 (1.83, 2.45) | *=0.41* |
| **Drinking** |  |  |  |
| Abstainers | 1.56 (1.30, 1.87) | 1.89 (1.58, 2.25) |  |
| Moderate drinkers | 1.00 | 1.00 |  |
| Heavy drinkers | 1.25 (1.02, 1.52) | 1.19 (1.01, 1.40) | *=0.73* |
| **Diet** |  |  |  |
| Healthy | 1.00 | 1.00 |  |
| Moderately healthy | 1.40 (1.19, 1.64) | 1.19 (1.00, 1.41) |  |
| Unhealthy | 2.17 (1.51, 3.11) | 2.06 (1.61, 2.63) | *=0.61* |
| **Physical activity** |  |  |  |
| Active | 1.00 | 1.00 |  |
| Moderately active | 1.06 (0.86, 1.30) | 1.23 (1.02, 1.48) |  |
| Inactive | 1.60 (1.35, 1.91) | 1.70 (1.45, 1.98) | *=0.50* |

HR=Hazard Ratios, CI=Confidence Interval

^a^ Model adjusted for age at baseline and sex

^b^ P for interaction between health behaviour and cohort
