# Supplementary material for: Health Behaviours, Socioeconomic Status, and Mortality: Further Analyses of the British Whitehall II and the French GAZEL Prospective Cohorts
Source: PLoS Med. 2011 Feb 22;8(2):e1000419. doi: 10.1371/journal.pmed.1000419 (PMC3043001; doi:10.1371/journal.pmed.1000419)
Supplement: Table S4 — Education. The role of health behaviours used as time-dependent covariates in explaining the association between education and all-cause mortality in the British Whitehall II (n = 9,754, deaths = 691) and the French GAZEL (n = 17,449, deaths = 881) cohort studies. (0.03 MB DOC) [file pmed.1000419.s004.doc]

Table S1 EDUCATION. Role of health behaviours used as time dependent covariates in explaining the association between education and all-cause mortality in the British Whitehall II (N=9754, Deaths=691) and the French GAZEL (N=17449, Deaths=881) cohort studies.

|  | **WHITEHALL II** | | **GAZEL** | |
| --- | --- | --- | --- | --- |
|  | **HR (95% CI)** | **%Δ ^c^** | **HR (95% CI)** | **%Δ ^c^** |
| Model 1^a^ | 1.43 (1.15, 1.79) |  | 1.56 (1.26, 1.91) |  |
| Model 1 + Smoking | 1.28 (1.02, 1.60) | 31 | 1.54 (1.25, 1.89) | 3 |
| Model 1 + Alcohol | 1.39 (1.11, 1.74) | 7 | 1.53 (1.24, 1.88) | 4 |
| Model 1 + Diet | 1.33 (1.06, 1.66) | 21 | 1.51 (1.23, 1.86) | 7 |
| Model 1 + Physical activity | 1.39 (1.11, 1.74) | 8 | 1.51 (1.23, 1.86) | 7 |
| Fully adjusted Model ^b^ | 1.17 (0.94, 1.47) | 56 | 1.44 (1.17, 1.78) | 17 |

HR=Hazard Ratio, CI=Confidence Interval

^a^ HR for lowest versus highest education adjusted for age at baseline and sex

^b^ HR for lowest versus highest education adjusted for age at baseline, sex, and all health behaviours

^c^ Percent attenuation in log HR= 100 x (β Model 1 - β Model 1+ health behaviour(s))/( β Model 1 ), where β=log(HR)
