# Supplementary material for: Health Behaviours, Socioeconomic Status, and Mortality: Further Analyses of the British Whitehall II and the French GAZEL Prospective Cohorts
Source: PLoS Med. 2011 Feb 22;8(2):e1000419. doi: 10.1371/journal.pmed.1000419 (PMC3043001; doi:10.1371/journal.pmed.1000419)
Supplement: Table S5 — Income. Sample characteristics of the British Whitehall II and the French GAZEL cohort studies. (0.03 MB DOC) [file pmed.1000419.s005.doc]

Table S5 INCOME. Sample characteristics of the British Whitehall II and the French GAZEL cohort studies.

|  | **Income** | | | **Overall** |
| --- | --- | --- | --- | --- |
|  | High | Intermediate | Low |  |
| **WHITEHALL II** |  |  |  |  |
| N (%) | 7405 (76.6) | 1662 (17.2) | 604 (6.3) | 9671 |
| Deaths (Rate^a^) | 497 (3.3) | 125 (4.2) | 67 (6.5) | 689 (3.6) |
| Mean age (SD) | 44.3 (6.0) | 44.1 (6.3) | 45.5 (6.2) | 44.4 (6.1) |
| **GAZEL** |  |  |  |  |
| N (%) | 1302 (7.6) | 11924 (69.6) | 3905 (22.8) | 17131 |
| Deaths (Rate^a^) | 48 (2.2) | 560 (2.8) | 262 (4.1) | 870 (3.1) |
| Mean age (SD) | 44.1 (3.7) | 43.3 (3.5) | 43.4 (3.5) | 43.4 (3.5) |

SD=Standard Deviation

^a^ Age- and sex-adjusted mortality rate per 1000 person-years

Note: In the Whitehall II study, income was not available at study baseline. We thus use a proxy measure composed of measures of car ownership and type of accommodation. The highest category represents participants owning a car and their house, the lowest represents participants not owning a car and living in rented accommodation. The intermediate category represents other combinations of car ownership and type of accommodation. In the GAZEL study income was measured at study baseline (1989) and the following three categories (based on quintiles of income, converted in Euro from French Francs) were used in the analysis: <1600€, 1600€ - 3800€, and ≥3800€.
