# Supplementary material for: Health Behaviours, Socioeconomic Status, and Mortality: Further Analyses of the British Whitehall II and the French GAZEL Prospective Cohorts
Source: PLoS Med. 2011 Feb 22;8(2):e1000419. doi: 10.1371/journal.pmed.1000419 (PMC3043001; doi:10.1371/journal.pmed.1000419)
Supplement: Table S6 — Income. The association of income with health behaviours in the British Whitehall II (n = 9,671 at first and n = 7,099 at last follow-up) and the French GAZEL (n = 17,131 at first and n = 14,859 at last follow-up) cohort studies. (0.03 MB DOC) [file pmed.1000419.s006.doc]

Table S6 INCOME. Association of income with health behaviours in the British Whitehall II (N=9671 at first and N=7099 at last follow-up) and the French GAZEL (N=17131 at first and N=14859 at last follow-up) cohort studies.

|  | **WHITEHALL II** | **GAZEL** | P*^b^* |
| --- | --- | --- | --- |
|  | **OR^a^ (95%CI)** | **OR^a^ (95%CI)** |  |
| **FIRST FOLLOW-UP** | | | |
| Smoking | 3.00 (2.56, 3.53) | 1.40 (1.22, 1.60) | *<0.001* |
| Heavy drinking | 1.14 (2.56, 3.53) | 1.08 (0.93, 1.25) | *=0.17* |
| Unhealthy diet | 3.97 (2.93, 5.38) | 1.65 (1.41, 1.94) | *<0.001* |
| Physically inactive | 4.71 (3.98, 5.59) | 2.30 (2.03, 2.59) | *<0.001* |
| **LAST FOLLOW-UP** | | | |
| Smoking | 3.25 (2.52, 4.19) | 1.15 (0.95, 1.40) | *<0.001* |
| Heavy drinking | 0.99 (0.78, 1.25) | 0.87 (0.75, 1.01) | *=0.08* |
| Unhealthy diet | 5.85 (3.70, 9.25) | 2.11 (1.54, 2.88) | *<0.001* |
| Physically inactive | 3.88 (3.21, 4.68) | 1.97 (1.74, 2.23) | *<0.001* |

OR=Odds Ratio; CI=Confidence Interval

^a^ Odds ratio for lowest versus highest income adjusted for age and sex

^b^ P for interaction between health behaviour and cohort
