# Supplementary material for: Health Behaviours, Socioeconomic Status, and Mortality: Further Analyses of the British Whitehall II and the French GAZEL Prospective Cohorts
Source: PLoS Med. 2011 Feb 22;8(2):e1000419. doi: 10.1371/journal.pmed.1000419 (PMC3043001; doi:10.1371/journal.pmed.1000419)
Supplement: Table S7 — Income. The association between health behaviours and all-cause mortality in the British Whitehall II (n = 9,671, deaths = 689) and the French GAZEL (n = 17,131, deaths = 870) cohort studies. (0.04 MB DOC) [file pmed.1000419.s007.doc]

Table S7 INCOME. The association between health behaviours and all-cause mortality in the British Whitehall II (N=9 671, Deaths=689) and the French GAZEL (N=17131, Deaths=870) cohort studies.

|  | **WHITEHALL II** | **GAZEL** | P^b^ |
| --- | --- | --- | --- |
|  | **HR** ^a^ **(95% CI)** | **HR** ^a^ **(95% CI)** |  |
| **Smoking** |  |  |  |
| Non smokers | 1.00 | 1.00 |  |
| Current smokers | 2.40 (2.00, 2.87) | 2.10 (1.81, 2.43) | *=0.41* |
| **Drinking** |  |  |  |
| Abstainers | 1.57 (1.31, 1.89) | 1.89 (1.58, 2.26) |  |
| Moderate drinkers | 1.00 | 1.00 |  |
| Heavy drinkers | 1.24 (1.01, 1.52) | 1.18 (1.00, 1.39) | *=0.73* |
| **Diet** |  |  |  |
| Healthy | 1.00 | 1.00 |  |
| Moderately healthy | 1.40 (1.19, 1.64) | 1.19 (1.00, 1.41) |  |
| Unhealthy | 2.18 (1.52, 3.12) | 2.07 (1.62, 2.65) | *=0.50* |
| **Physical activity** |  |  |  |
| Active | 1.00 | 1.00 |  |
| Moderately active | 1.06 (0.86, 1.30) | 1.23 (1.02, 1.49) |  |
| Inactive | 1.61 (1.35, 1.92) | 1.69 (1.45, 1.98) | *=0.61* |

HR=Hazard Ratios, CI=Confidence Interval

^a^ Model adjusted for age at baseline and sex

^b^ P for interaction between health behaviour and cohort
