# Supplementary material for: Health Behaviours, Socioeconomic Status, and Mortality: Further Analyses of the British Whitehall II and the French GAZEL Prospective Cohorts
Source: PLoS Med. 2011 Feb 22;8(2):e1000419. doi: 10.1371/journal.pmed.1000419 (PMC3043001; doi:10.1371/journal.pmed.1000419)
Supplement: Table S8 — Income. The role of health behaviours used as time-dependent covariates in explaining the association between income and all-cause mortality in the British Whitehall II (n = 9,671, deaths = 689) and the French GAZEL (n = 17,131, deaths = 870) cohort studies. (0.03 MB DOC) [file pmed.1000419.s008.doc]

Table S8 INCOME. Role of health behaviours used as time dependent covariates in explaining the association between income and all-cause mortality in the British Whitehall II (N=9 671, Deaths=689) and the French GAZEL (N=17 131, Deaths=870) cohort studies.

|  | **WHITEHALL II** | | **GAZEL** | |
| --- | --- | --- | --- | --- |
|  | **HR (95% CI)** | **%Δ ^c^** | **HR (95% CI)** | **%Δ ^c^** |
| Model 1^a^ | 1.90 (1.49, 2.41) |  | 2.05 (1.60, 2.63) |  |
| Model 1 + Smoking | 1.60 (1.26, 2.04) | 26 | 1.99 (1.56, 2.55) | 4 |
| Model 1 + Alcohol | 1.78 (1.40, 2.26) | 10 | 1.95 (1.52, 2.50) | 7 |
| Model 1 + Diet | 1.71 (1.34, 2.19) | 16 | 1.98 (1.54, 2.53) | 5 |
| Model 1 + Physical activity | 1.68 (1.31, 2.14) | 19 | 1.90 (1.49, 2.44) | 10 |
| Fully adjusted Model ^b^ | 1.32 (1.03, 1.70) | 56 | 1.74 (1.36, 2.23) | 23 |

HR=Hazard Ratios, CI=Confidence Interval

^a^ HR for lowest versus highest income adjusted for age at baseline and sex

^b^ HR for lowest versus highest income adjusted for age at baseline, sex, and all health behaviours

^c^ Percent attenuation in log HR= 100 x (β Model 1 - β Model 1+ health behaviour(s))/( β Model 1 ), where β=log(HR)
