# Supplementary material for: Health Behaviours, Socioeconomic Status, and Mortality: Further Analyses of the British Whitehall II and the French GAZEL Prospective Cohorts
Source: PLoS Med. 2011 Feb 22;8(2):e1000419. doi: 10.1371/journal.pmed.1000419 (PMC3043001; doi:10.1371/journal.pmed.1000419)
Supplement: Table S9 — GAZEL white-collar workers. Sample characteristics of the British Whitehall II and the French GAZEL cohort studies. (0.03 MB DOC) [file pmed.1000419.s009.doc]

Table S9 GAZEL WHITE COLLAR WORKERS. Sample characteristics of the British Whitehall II and the French GAZEL cohort studies.

|  | **Occupational position** | | | **Overall** |
| --- | --- | --- | --- | --- |
|  | High | Intermediate | Low |  |
| **WHITEHALL II** |  |  |  |  |
| N (%) | 2914 (29.8) | 4744 (48.6) | 2113 (21.6) | 9771 |
| Deaths (Rate^a^) | 197 (3.1) | 322 (3.8) | 174 (5.2) | 693 (3.6) |
| Mean age (SD) | 45.0 (5.8) | 43.3 (6.0) | 46.0 (6.0) | 44.4 (6.1) |
| **GAZEL** |  |  |  |  |
| N (%) | 1988 (24.6) | 4499 (55.7) | 1592 (19.7) | 8079 |
| Deaths (Rate^a^) | 86 (2.3) | 184 (2.7) | 80 (4.2) | 350 (2.6) |
| Mean age (SD) | 44.1 (3.3) | 42.7 (3.9) | 41.5 (4.1) | 44.3 (3.5) |

SD=Standard Deviation

^a^ Age- and sex-adjusted mortality rate per 1000 person-years
