# Supplementary material for: Health Behaviours, Socioeconomic Status, and Mortality: Further Analyses of the British Whitehall II and the French GAZEL Prospective Cohorts
Source: PLoS Med. 2011 Feb 22;8(2):e1000419. doi: 10.1371/journal.pmed.1000419 (PMC3043001; doi:10.1371/journal.pmed.1000419)
Supplement: Table S10 — GAZEL white-collar workers. The association of occupational position with health behaviours in the British Whitehall II cohort (n = 9,771 at first and n = 7,166 at last follow-up) and in white-collar workers of the French GAZEL cohort (n = 8,079 at first and n = 6,902 at last follow-up). (0.03 MB DOC) [file pmed.1000419.s010.doc]

Table S10 GAZEL WHITE COLLAR WORKERS. Association of occupational position with health behaviours in the British Whitehall II cohort (N=9771 at first and N=7166 at last follow-up) and in white-collar workers of the French GAZEL cohort (N=8079 at first and N=6902 at last follow-up).

|  | **WHITEHALL II** | **GAZEL** | P^b^ |
| --- | --- | --- | --- |
|  | **OR^a^ (95%CI)** | **OR^a^ (95%CI)** |  |
| **FIRST FOLLOW-UP** | | | |
| Smoking | 3.68 (3.11, 4.36) | 1.25 (1.05, 1.49) | *<0.001* |
| Heavy drinking | 0.50 (0.42, 0.60) | 0.89 (0.72, 1.09) | *0.24* |
| Unhealthy diet | 7.42 (5.19, 10.60) | 1.32 (1.08, 1.62) | *<0.001* |
| Physically inactive | 6.07 (5.00, 7.36) | 1.99 (1.71, 2.32) | *<0.001* |
| **LAST FOLLOW-UP** | | | |
| Smoking | 4.17 (3.17, 5.47) | 1.12 (0.88, 1.43) | *<0.001* |
| Heavy drinking | 0.36 (0.30, 0.44) | 0.83 (0.67, 1.02) | *0.004* |
| Unhealthy diet | 9.99 (5.66, 17.63) | 2.60 (1.74, 3.88) | *<0.001* |
| Physically inactive | 2.27 (1.92, 2.70) | 1.75 (1.74, 3.88) | *<0.001* |

OR=Odds Ratio; CI=Confidence Interval

^a^ Odds Ratio for lowest versus highest occupational position adjusted for age and sex

^b^ P for interaction between health behaviour and cohort
