# Supplementary material for: Health Behaviours, Socioeconomic Status, and Mortality: Further Analyses of the British Whitehall II and the French GAZEL Prospective Cohorts
Source: PLoS Med. 2011 Feb 22;8(2):e1000419. doi: 10.1371/journal.pmed.1000419 (PMC3043001; doi:10.1371/journal.pmed.1000419)
Supplement: Table S11 — GAZEL white-collar workers. The association between health behaviours and all-cause mortality in the British Whitehall II cohort (n = 9,771, deaths = 693) and in white-collar workers of the French GAZEL cohort (n = 8,079, deaths = 350). (0.04 MB DOC) [file pmed.1000419.s011.doc]

Table S11 GAZEL WHITE COLLAR WORKERS. The association between health behaviours and all-cause mortality in the British Whitehall II cohort (N=9771, Deaths=693) and in white-collar workers of the French GAZEL cohort (N=8079, Deaths=350).

|  | **WHITEHALL II** | **GAZEL** | P^b^ |
| --- | --- | --- | --- |
|  | **HR** ^a^ **(95% CI)** | **HR** ^a^ **(95% CI)** |  |
| **Smoking** |  |  |  |
| Non smokers | 1.00 | 1.00 |  |
| Current smokers | 2.38 (1.99, 2.85) | 2.21 (1.75, 2.79) | *0.41* |
| **Drinking** |  |  |  |
| Abstainers | 1.56 (1.30, 1.87) | 1.94 (1.49, 2.52) |  |
| Moderate drinkers | 1.00 | 1.00 |  |
| Heavy drinkers | 1.25 (1.02, 1.52) | 1.24 (0.95, 1.63) | *0.73* |
| **Diet** |  |  |  |
| Healthy | 1.00 | 1.00 |  |
| Moderately healthy | 1.41 (1.20, 1.65) | 1.27 (0.97, 1.66) |  |
| Unhealthy | 2.14 (1.49, 3.07) | 2.40 (1.64, 3.52) | *0.61* |
| **Physical activity** |  |  |  |
| Active | 1.00 | 1.00 |  |
| Moderately active | 1.05 (1.49, 3.07) | 1.28 (0.95, 1.73) |  |
| Inactive | 1.60 (1.34, 1.90) | 1.67 (1.31, 2.14) | *0.50* |

HR=Hazard Ratios, CI=Confidence Interval

^a^ Model adjusted for age at baseline and sex

^b^ P for interaction between health behaviour and cohort
