# Supplementary material for: Health Behaviours, Socioeconomic Status, and Mortality: Further Analyses of the British Whitehall II and the French GAZEL Prospective Cohorts
Source: PLoS Med. 2011 Feb 22;8(2):e1000419. doi: 10.1371/journal.pmed.1000419 (PMC3043001; doi:10.1371/journal.pmed.1000419)
Supplement: Table S12 — GAZEL white-collar workers. The role of health behaviours used as time-dependent covariates in explaining the association between occupational position and all-cause mortality in the British Whitehall II cohort (n = 9,771, deaths = 693) and in white-collar workers of the French GAZEL cohort (n = 8,079, deaths = 350). (0.03 MB DOC) [file pmed.1000419.s012.doc]

Table S12 GAZEL WHITE COLLAR WORKERS. Role of health behaviours used as time dependent covariates in explaining the association between occupational position and all-cause mortality in the British Whitehall II cohort (N=9771, Deaths=693) and in white-collar workers of the French GAZEL cohort (N=8079, Deaths=350).

|  | **WHITEHALL II** | | **GAZEL** | |
| --- | --- | --- | --- | --- |
|  | **HR (95% CI)** | **%Δ ^c^** | **HR (95% CI)** | **%Δ ^c^** |
| Model 1^a^ | 1.62 (1.28, 2.05) |  | 2.26 (1.63, 3.13) |  |
| Model 1 + Smoking | 1.39 (1.09, 1.75) | 32 | 2.20 (1.59, 3.05) | 3 |
| Model 1 + Alcohol | 1.52 (1.19, 1.93) | 14 | 2.14 (1.54, 2.96) | 7 |
| Model 1 + Diet | 1.44 (1.13, 1.83) | 25 | 2.19 (1.58, 3.04) | 4 |
| Model 1 + Physical activity | 1.47 (1.16, 1.86) | 21 | 2.16 (1.56, 3.00) | 5 |
| Fully adjusted Model ^b^ | 1.13 (0.88, 1.44) | 75 | 1.96 (1.41, 2.73) | 17 |

HR=Hazard Ratios, CI=Confidence Interval

^a^ HR for lowest versus highest occupational position adjusted for age at baseline and sex

^b^ HR for lowest versus highest occupational position adjusted for age at baseline, sex, and all health behaviours

^c^ Percent attenuation in log HR= 100 x (β Model 1 - β Model 1+ health behaviour(s))/( β Model 1 ), where β=log(HR)
