# Supplementary material for: Health Behaviours, Socioeconomic Status, and Mortality: Further Analyses of the British Whitehall II and the French GAZEL Prospective Cohorts
Source: PLoS Med. 2011 Feb 22;8(2):e1000419. doi: 10.1371/journal.pmed.1000419 (PMC3043001; doi:10.1371/journal.pmed.1000419)
Supplement: Table S13 — Absolute probabilities and absolute differences in probabilities. The association between health behaviours and all-cause mortality in the British Whitehall II (n = 9,771, deaths = 693) and the French GAZEL (n = 17,760, deaths = 908) cohort studies. (0.05 MB DOC) [file pmed.1000419.s013.doc]

Table S13 and S14 – ABSOLUTE PROBABILITIES AND ABSOLUTE DIFFERENCES IN PROBABILITIES

Table S13. The association between health behaviours and all-cause mortality in the British Whitehall II (N=9 771, Deaths=693) and the French GAZEL (N=17 760, Deaths=908) cohort studies.

|  | **WHITEHALL II** | | | **GAZEL** | | | P^c^ |
| --- | --- | --- | --- | --- | --- | --- | --- |
|  | **Mortality rate ^a^** | **Rate Δ^a^(95% CI)** | **HR** ^b^ **(95% CI)** | **Mortality rate ^a^** | **Rate Δ^a^(95% CI)** | **HR** ^b^ **(95% CI)** |  |
| **Smoking** |  |  |  |  |  |  |  |
| Non smokers | 3.2 | - | 1.00 | 2.7 | - | 1.00 |  |
| Current smokers | 6.2 | 3.0 (0.4, 5.5) | 2.38 (1.99, 2.85) | 5.1 | 2.4 (0.8, 4.0) | 2.11 (1.82, 2.44) | *0.36* |
| **Drinking** |  |  |  |  |  |  |  |
| Abstainers | 4.9 | 1.8 (-0.6, 4.2) | 1.56 (1.99, 2.85) | 4.6 | 1.8 (0.0, 3.6) | 1.89 (1.59, 2.24) |  |
| Moderate drinkers | 3.1 | - | 1.00 | 2.7 | - | 1.00 |  |
| Heavy drinkers | 4.1 | 1.0 (-1.4, 3.4) | 1.25 (1.02, 1.52) | 3.2 | 0.5 (-1.1, 2.1) | 1.14 (0.97, 1.34) | *0.70* |
| **Diet** |  |  |  |  |  |  |  |
| Healthy | 3.4 | - | 1.00 | 2.8 | - | 1.00 |  |
| Moderately healthy | 4.0 | 0.6 (-1.3, 2.6) | 1.41 (1.20, 1.65) | 3.0 | 0.2 (-1.4, 1.7) | 1.17 (0.99, 1.38) |  |
| Unhealthy | 5.8 | 2.4 (-2.8, 7.7) | 2.14 (1.49, 3.07) | 4.4 | 1.6 (-0.9, 4.0) | 2.04 (1.61, 2.60) | *0.49* |
| **Physical activity** |  |  |  |  |  |  |  |
| Active | 3.2 | - | 1.00 | 2.4 | - | 1.00 |  |
| Moderately active | 3.1 | 0.0 (-2.3, 2.2) | 1.05 (0.86, 1.30) | 2.7 | 0.3 (-1.3, 1.9) | 1.22 (1.01, 1.47) |  |
| Inactive | 5.4 | 2.2 (-0.1, 4.5) | 1.60 (1.34, 1.90) | 4.1 | 1.7 (0.2, 3.1) | 1.67 (1.43, 1.95) | *0.45* |

Δ=Difference; HR=Hazard Ratios, CI=Confidence Interval

^a^ Age- and sex- adjusted rate and rate differences calculated using additive models

^b^ Cox regression model adjusted for age at baseline and sex

^c^ P for interaction between health behaviour and cohort, Cox regression
