# Supplementary material for: Health Behaviours, Socioeconomic Status, and Mortality: Further Analyses of the British Whitehall II and the French GAZEL Prospective Cohorts
Source: PLoS Med. 2011 Feb 22;8(2):e1000419. doi: 10.1371/journal.pmed.1000419 (PMC3043001; doi:10.1371/journal.pmed.1000419)
Supplement: Table S14 — Absolute probabilities and absolute differences in probabilities. The role of health behaviours used as time-dependent covariates in explaining the association between occupational position and all-cause mortality in the British Whitehall II (n = 9,771, deaths = 693) and the French GAZEL (n = 17,760, deaths = 908) cohort studies. (0.04 MB DOC) [file pmed.1000419.s014.doc]

**Table S13-14 – ABSOLUTE PROBABILITIES AND ABSOLUTE DIFFERENCES IN PROBABILITIES**

Table S14 Role of health behaviours used as time dependent covariates in explaining the association between occupational position and all-cause mortality in the British Whitehall II (N=9 771, Deaths=693) and the French GAZEL (N=17 760, Deaths=908) cohort studies.

|  | **Rate Δ^c^(95% CI)** | **% attenuation^d^** | **HR^e^ (95% CI)** | **% attenuation^f^ (95%CI)** |
| --- | --- | --- | --- | --- |
| **WHITEHALL II** |  |  |  |  |
| Model 1 (SES, age- and sex- adjusted)^a^ | 1.5 (-1.3, 4.3) |  | 1.62 (1.28, 2.05) |  |
| Model 1 + Smoking | 1.0 (-1.8, 3.9) | 31 | 1.39 (1.09, 1.75) | 32 (20 - 62) |
| Model 1 + Alcohol | 1.2 (-1.6, 4.1) | 18 | 1.52 (1.19, 1.93) | 14 (3 - 37) |
| Model 1 + Diet | 1.3 (-1.6, 4.1) | 16 | 1.44 (1.13, 1.83) | 25 (12 - 55) |
| Model 1 + Physical activity | 1.1 (-1.7, 3.9) | 27 | 1.47 (1.16, 1.86) | 21 (11 - 43) |
| Fully adjusted Model ^b^ | 0.3 (-2.6, 3.3) | 78 | 1.13 (0.88, 1.44) | 75 (44 - 149) |
| **GAZEL** |  |  |  |  |
| Model 1 (SES, age- and sex- adjusted)^a^ | 2.1 (0.1, 4.1) |  | 1.94 (1.58, 2.39) |  |
| Model 1 + Smoking | 2.0 (0.0, 4.0) | 4 | 1.89 (1.51, 2.28) | 4 (2 - 8) |
| Model 1 + Alcohol | 1.9 (-0.1, 4.0) | 6 | 1.85 (1.51, 2.28) | 7 (4 - 11) |
| Model 1 + Diet | 2.0 (0.0, 4.0) | 2 | 1.89 (1.54, 2.33) | 4 (2 - 8) |
| Model 1 + Physical activity | 1.9 (-0.1, 3.9) | 9 | 1.85 (1.50, 2.27) | 8 (4 - 12) |
| Fully adjusted Model ^b^ | 1.7 (-0.3, 3.7) | 19 | 1.71 (1.39, 2.10) | 19 (13 - 29) |

D= Difference; HR=Hazard Ratios; CI=Confidence Interval

^a^ Lowest versus highest occupational position, age- and sex- adjusted

^b^ Lowest versus highest occupational position adjusted for age , sex, and all health behaviours

^c^ Difference in mortality rate per 1000 person-years between lowest and highest occupational position

^d^ Percent attenuation in rate difference=100 x (bModel 1 -bModel 1 + health behaviour(s))/(b Model 1 )

^e^ HR for lowest versus highest occupational position, age- and sex- adjusted

^f^ Percent attenuation in log HR= 100 x (β Model 1 - β Model 1+ health behaviour(s))/( β Model 1 ), where β=log(HR)

^g^ Bias corrected accelerated bootstrap 95% confidence interval for percent attenuation in HR associated to occupational position
