# Supplementary material for: Health Behaviours, Socioeconomic Status, and Mortality: Further Analyses of the British Whitehall II and the French GAZEL Prospective Cohorts
Source: PLoS Med. 2011 Feb 22;8(2):e1000419. doi: 10.1371/journal.pmed.1000419 (PMC3043001; doi:10.1371/journal.pmed.1000419)
Supplement: Table S15 — Inverse probability weighted. Association of occupational position with health behaviours in the British Whitehall II (n = 9,771 at first and n = 7,166 at last follow-up) and the French GAZEL (n = 17,760 at first and n = 15,377 at last follow-up) cohort studies. (0.03 MB DOC) [file pmed.1000419.s015.doc]

**Table S15 and S16 – INVERSE PROBABILITY WEIGHTED**

Table S15 INVERSE PROBABILITY WEIGHTED. Association of occupational position with health behaviours in the British Whitehall II (N=9771 at first and N=7166 &t last follow-up) and the French GAZEL (N=17760 at first and N=15377 at last follow-up) cohort studies.

|  | **WHITEHALL II** | **GAZEL** | P^b^ |
| --- | --- | --- | --- |
|  | **OR^a^ (95% CI)** | **OR^a^ (95% CI)** |  |
| **FIRST FOLLOW-UP** | | | |
| Smoking | 3.67 (3.09, 4.36) | 1.31 (1.17, 1.47) | *<0.001* |
| Heavy drinking | 0.49 (0.41, 0.59) | 1.13 (1.00, 1.27) | *<0.001* |
| Unhealthy diet | 7.42 (5.20, 10.57) | 1.30 (1.14, 1.48) | *<0.001* |
| Physically inactive | 6.10 (4.94, 7.53) | 1.94 (1.75, 2.15) | *<0.001* |
| **LAST FOLLOW-UP** | | | |
| Smoking | 4.16 (3.12, 5.55) | 1.14 (0.97, 1.34) | *<0.001* |
| Heavy drinking | 0.36 (0.29, 0.44) | 0.90 (0.79, 1.02) | *<0.001* |
| Unhealthy diet | 10.12 (5.50, 18.63) | 1.89 (1.46, 2.44) | *<0.001* |
| Physically inactive | 2.29 (1.91, 2.73) | 1.63 (1.47, 1.81) | *<0.001* |

OR=Odds Ratio; CI=Confidence Interval

^a^ Odds ratio for lowest versus highest occupational position adjusted for age and sex

^b^ P for interaction between health behaviour and cohort
