# Supplementary material for: Health Behaviours, Socioeconomic Status, and Mortality: Further Analyses of the British Whitehall II and the French GAZEL Prospective Cohorts
Source: PLoS Med. 2011 Feb 22;8(2):e1000419. doi: 10.1371/journal.pmed.1000419 (PMC3043001; doi:10.1371/journal.pmed.1000419)
Supplement: Table S16 — Inverse probability weighted. Role of health behaviours used as time-dependent covariates in explaining the association between occupational position and all-cause mortality in the British Whitehall II (n = 9,771, deaths = 693) and the French GAZEL (n = 17,760, deaths = 908) cohort studies. (0.03 MB DOC) [file pmed.1000419.s016.doc]

**Table S15 and S16 – INVERSE PROBABILITY WEIGHTED**

Table S16. INVERSE PROBABILITY WEIGHTED. Role of health behaviours used as time dependent covariates in explaining the association between occupational position and all-cause mortality in the British Whitehall II (N=9 771, Deaths=693) and the French GAZEL (N=17 760, Deaths=908) cohort studies.

|  | **WHITEHALL II** | | **GAZEL** | |
| --- | --- | --- | --- | --- |
|  | **HR (95% CI)** | **%Δ ^c^** | **HR** | **%Δ ^c^** |
| Model 1^a^ | 1.68 (1.36, 2.08) |  | 1.98 (1.63, 2.42) |  |
| Model 1 + Smoking | 1.42 (1.12, 1.80) | 32 | 1.90 (1.55, 2.33) | 6 |
| Model 1 + Alcohol | 1.56 (1.22, 2.00) | 15 | 1.86 (1.52, 2.28) | 10 |
| Model 1 + Diet | 1.45 (1.13, 1.85) | 29 | 1.88 (1.53, 2.32) | 8 |
| Model 1 + Physical activity | 1.47 (1.16, 1.88) | 26 | 1.87 (1.52, 2.30) | 9 |
| Fully adjusted Model ^b^ | 1.12 (0.87, 1.45) | 78 | 1.71 (1.38, 2.11) | 22 |

HR=Hazard Ratios, CI=Confidence Interval

^a^ HR for lowest versus highest occupational position adjusted for age at baseline and sex

^b^ HR for lowest versus highest occupational position adjusted for age at baseline, sex, and all health behaviours

^c^ Percent attenuation in log HR= 100 x (β Model 1 - β Model 1+ health behaviour(s))/( β Model 1 ), where β=log(HR)
