# Supplementary material for: Health Behaviours, Socioeconomic Status, and Mortality: Further Analyses of the British Whitehall II and the French GAZEL Prospective Cohorts
Source: PLoS Med. 2011 Feb 22;8(2):e1000419. doi: 10.1371/journal.pmed.1000419 (PMC3043001; doi:10.1371/journal.pmed.1000419)
Supplement: Text S1 — Results from supplementary analyses. Tables S1–S16 in a single file for reader's convenience. (0.29 MB DOC) [file pmed.1000419.s017.doc]

**Results from supplementary analyses**

**Tables S1 to S4– EDUCATION**

Table S1 EDUCATION. Sample characteristics of the British Whitehall II and the French GAZEL cohort studies.

|  | **Education** | | | **Overall** |
| --- | --- | --- | --- | --- |
|  | Tertiary | Secondary | Primary |  |
| **WHITEHALL II** |  |  |  |  |
| N (%) | 2430 (24.9) | 5232 (53.6) | 2092 (21.5) | 9 754 |
| Deaths (Rate^a^) | 105 (2.3) | 385 (4.5) | 201 (3.2) | 691 (3.6) |
| Mean age (SD) | 42.6 (5.8) | 43.3 (5.6) | 49.0 (5.1) | 44.4 (6.1) |
| **GAZEL** |  |  |  |  |
| N (%) | 3448 (19.8) | 10299 (59.0) | 3702 (21.2) | 17449 |
| Deaths (Rate^a^) | 145 (2.5) | 513 (3.1) | 223 (3.8) | 881 (3.1) |
| Mean age (SD) | 43.4 (3.5) | 43.2 (3.5) | 43.8 (3.5) | 43.4 (3.5) |

SD=Standard Deviation

^a^ Age- and sex-adjusted mortality rate per 1000 person-years

Note: In Whitehall II, education categorized as university, secondary and primary education was collected at Phase 5 (1997/99) and was available on 6776 participants. The remaining participants N=2978 were imputed using multiple imputation.

Table S1 EDUCATION. Association of education with health behaviours in the British Whitehall II (N=9754 at first and N=7163 at last follow-up) and the French GAZEL (N=17449 at first and N=15130 at last follow-up) cohort studies.

|  | **WHITEHALL II** | **GAZEL** | P^b^ |
| --- | --- | --- | --- |
|  | **OR^a^ (95%CI)** | **OR^a^ (95%CI)** |  |
| **FIRST FOLLOW-UP** | | | |
| Smoking | 3.85 (3.22, 4.61) | 1.21 (1.08, 1.35) | *<0.001* |
| Heavy drinking | 0.80 (0.66, 0.95) | 1.15 (1.02, 1.30) | *<0.001* |
| Unhealthy diet | 3.80 (2.62, 5.51) | 1.57 (1.38, 1.80) | *<0.001* |
| Physically inactive | 2.73 (2.62, 5.51) | 1.62 (1.47, 1.80) | *<0.001* |
| **LAST FOLLOW-UP** | | | |
| Smoking | 2.86 (2.21, 3.70) | 0.99 (0.85, 1.16) | *<0.001* |
| Heavy drinking | 0.62 (0.52, 0.74) | 1.06 (0.93, 1.20) | *<0.001* |
| Unhealthy diet | 3.00 (1.79, 5.06) | 1.75 (1.35, 2.28) | *=0.02* |
| Physically inactive | 1.22 (1.04, 1.43) | 1.44 (1.30, 1.60) | *=0.02* |

OR=Odds Ratio; CI=Confidence Interval

^a^ Odds ratio for lowest versus highest education, adjusted for age and sex

^b^ P for interaction between health behaviour and cohort

Table S2 EDUCATION. The association between health behaviours and all-cause mortality in the British Whitehall II (N=9754, Deaths=691) and the French GAZEL (N=17449, Deaths=881) cohort studies.

|  | **WHITEHALL II** | **GAZEL** | P^b^ |
| --- | --- | --- | --- |
|  | **HR** ^a^ **(95% CI)** | **HR** ^a^ **(95% CI)** |  |
| **Smoking** |  |  |  |
| Non smokers | 1.00 | 1.00 |  |
| Current smokers | 2.38 (1.99, 2.85) | 2.12 (1.83, 2.45) | *=0.41* |
| **Drinking** |  |  |  |
| Abstainers | 1.56 (1.30, 1.87) | 1.89 (1.58, 2.25) |  |
| Moderate drinkers | 1.00 | 1.00 |  |
| Heavy drinkers | 1.25 (1.02, 1.52) | 1.19 (1.01, 1.40) | *=0.73* |
| **Diet** |  |  |  |
| Healthy | 1.00 | 1.00 |  |
| Moderately healthy | 1.40 (1.19, 1.64) | 1.19 (1.00, 1.41) |  |
| Unhealthy | 2.17 (1.51, 3.11) | 2.06 (1.61, 2.63) | *=0.61* |
| **Physical activity** |  |  |  |
| Active | 1.00 | 1.00 |  |
| Moderately active | 1.06 (0.86, 1.30) | 1.23 (1.02, 1.48) |  |
| Inactive | 1.60 (1.35, 1.91) | 1.70 (1.45, 1.98) | *=0.50* |

HR=Hazard Ratios, CI=Confidence Interval

^a^ Model adjusted for age at baseline and sex

^b^ P for interaction between health behaviour and cohort

Table S3 EDUCATION. Role of health behaviours used as time dependent covariates in explaining the association between education and all-cause mortality in the British Whitehall II (N=9754, Deaths=691) and the French GAZEL (N=17449, Deaths=881) cohort studies.

|  | **WHITEHALL II** | | **GAZEL** | |
| --- | --- | --- | --- | --- |
|  | **HR (95% CI)** | **%Δ ^c^** | **HR (95% CI)** | **%Δ ^c^** |
| Model 1^a^ | 1.43 (1.15, 1.79) |  | 1.56 (1.26, 1.91) |  |
| Model 1 + Smoking | 1.28 (1.02, 1.60) | 31 | 1.54 (1.25, 1.89) | 3 |
| Model 1 + Alcohol | 1.39 (1.11, 1.74) | 7 | 1.53 (1.24, 1.88) | 4 |
| Model 1 + Diet | 1.33 (1.06, 1.66) | 21 | 1.51 (1.23, 1.86) | 7 |
| Model 1 + Physical activity | 1.39 (1.11, 1.74) | 8 | 1.51 (1.23, 1.86) | 7 |
| Fully adjusted Model ^b^ | 1.17 (0.94, 1.47) | 56 | 1.44 (1.17, 1.78) | 17 |

HR=Hazard Ratio, CI=Confidence Interval

^a^ HR for lowest versus highest education adjusted for age at baseline and sex

^b^ HR for lowest versus highest education adjusted for age at baseline, sex, and all health behaviours

^c^ Percent attenuation in log HR= 100 x (β Model 1 - β Model 1+ health behaviour(s))/( β Model 1 ), where β=log(HR)

**Tables S5 to S8 – INCOME**

Table S5 INCOME. Sample characteristics of the British Whitehall II and the French GAZEL cohort studies.

|  | **Income** | | | **Overall** |
| --- | --- | --- | --- | --- |
|  | High | Intermediate | Low |  |
| **WHITEHALL II** |  |  |  |  |
| N (%) | 7405 (76.6) | 1662 (17.2) | 604 (6.3) | 9671 |
| Deaths (Rate^a^) | 497 (3.3) | 125 (4.2) | 67 (6.5) | 689 (3.6) |
| Mean age (SD) | 44.3 (6.0) | 44.1 (6.3) | 45.5 (6.2) | 44.4 (6.1) |
| **GAZEL** |  |  |  |  |
| N (%) | 1302 (7.6) | 11924 (69.6) | 3905 (22.8) | 17131 |
| Deaths (Rate^a^) | 48 (2.2) | 560 (2.8) | 262 (4.1) | 870 (3.1) |
| Mean age (SD) | 44.1 (3.7) | 43.3 (3.5) | 43.4 (3.5) | 43.4 (3.5) |

SD=Standard Deviation

^a^ Age- and sex-adjusted mortality rate per 1000 person-years

Note: In the Whitehall II study, income was not available at study baseline. We thus use a proxy measure composed of measures of car ownership and type of accommodation. The highest category represents participants owning a car and their house, the lowest represents participants not owning a car and living in rented accommodation. The intermediate category represents other combinations of car ownership and type of accommodation. In the GAZEL study income was measured at study baseline (1989) and the following three categories (based on quintiles of income, converted in Euro from French Francs) were used in the analysis: <1600€, 1600€ - 3800€, and ≥3800€.

Table S6 INCOME. Association of income with health behaviours in the British Whitehall II (N=9671 at first and N=7099 at last follow-up) and the French GAZEL (N=17131 at first and N=14859 at last follow-up) cohort studies.

|  | **WHITEHALL II** | **GAZEL** | P*^b^* |
| --- | --- | --- | --- |
|  | **OR^a^ (95%CI)** | **OR^a^ (95%CI)** |  |
| **FIRST FOLLOW-UP** | | | |
| Smoking | 3.00 (2.56, 3.53) | 1.40 (1.22, 1.60) | *<0.001* |
| Heavy drinking | 1.14 (2.56, 3.53) | 1.08 (0.93, 1.25) | *=0.17* |
| Unhealthy diet | 3.97 (2.93, 5.38) | 1.65 (1.41, 1.94) | *<0.001* |
| Physically inactive | 4.71 (3.98, 5.59) | 2.30 (2.03, 2.59) | *<0.001* |
| **LAST FOLLOW-UP** | | | |
| Smoking | 3.25 (2.52, 4.19) | 1.15 (0.95, 1.40) | *<0.001* |
| Heavy drinking | 0.99 (0.78, 1.25) | 0.87 (0.75, 1.01) | *=0.08* |
| Unhealthy diet | 5.85 (3.70, 9.25) | 2.11 (1.54, 2.88) | *<0.001* |
| Physically inactive | 3.88 (3.21, 4.68) | 1.97 (1.74, 2.23) | *<0.001* |

OR=Odds Ratio; CI=Confidence Interval

^a^ Odds ratio for lowest versus highest income adjusted for age and sex

^b^ P for interaction between health behaviour and cohort

Table S7 INCOME. The association between health behaviours and all-cause mortality in the British Whitehall II (N=9 671, Deaths=689) and the French GAZEL (N=17131, Deaths=870) cohort studies.

|  | **WHITEHALL II** | **GAZEL** | P^b^ |
| --- | --- | --- | --- |
|  | **HR** ^a^ **(95% CI)** | **HR** ^a^ **(95% CI)** |  |
| **Smoking** |  |  |  |
| Non smokers | 1.00 | 1.00 |  |
| Current smokers | 2.40 (2.00, 2.87) | 2.10 (1.81, 2.43) | *=0.41* |
| **Drinking** |  |  |  |
| Abstainers | 1.57 (1.31, 1.89) | 1.89 (1.58, 2.26) |  |
| Moderate drinkers | 1.00 | 1.00 |  |
| Heavy drinkers | 1.24 (1.01, 1.52) | 1.18 (1.00, 1.39) | *=0.73* |
| **Diet** |  |  |  |
| Healthy | 1.00 | 1.00 |  |
| Moderately healthy | 1.40 (1.19, 1.64) | 1.19 (1.00, 1.41) |  |
| Unhealthy | 2.18 (1.52, 3.12) | 2.07 (1.62, 2.65) | *=0.50* |
| **Physical activity** |  |  |  |
| Active | 1.00 | 1.00 |  |
| Moderately active | 1.06 (0.86, 1.30) | 1.23 (1.02, 1.49) |  |
| Inactive | 1.61 (1.35, 1.92) | 1.69 (1.45, 1.98) | *=0.61* |

HR=Hazard Ratios, CI=Confidence Interval

^a^ Model adjusted for age at baseline and sex

^b^ P for interaction between health behaviour and cohort

Table S8 INCOME. Role of health behaviours used as time dependent covariates in explaining the association between income and all-cause mortality in the British Whitehall II (N=9 671, Deaths=689) and the French GAZEL (N=17 131, Deaths=870) cohort studies.

|  | **WHITEHALL II** | | **GAZEL** | |
| --- | --- | --- | --- | --- |
|  | **HR (95% CI)** | **%Δ ^c^** | **HR (95% CI)** | **%Δ ^c^** |
| Model 1^a^ | 1.90 (1.49, 2.41) |  | 2.05 (1.60, 2.63) |  |
| Model 1 + Smoking | 1.60 (1.26, 2.04) | 26 | 1.99 (1.56, 2.55) | 4 |
| Model 1 + Alcohol | 1.78 (1.40, 2.26) | 10 | 1.95 (1.52, 2.50) | 7 |
| Model 1 + Diet | 1.71 (1.34, 2.19) | 16 | 1.98 (1.54, 2.53) | 5 |
| Model 1 + Physical activity | 1.68 (1.31, 2.14) | 19 | 1.90 (1.49, 2.44) | 10 |
| Fully adjusted Model ^b^ | 1.32 (1.03, 1.70) | 56 | 1.74 (1.36, 2.23) | 23 |

HR=Hazard Ratios, CI=Confidence Interval

^a^ HR for lowest versus highest income adjusted for age at baseline and sex

^b^ HR for lowest versus highest income adjusted for age at baseline, sex, and all health behaviours

^c^ Percent attenuation in log HR= 100 x (β Model 1 - β Model 1+ health behaviour(s))/( β Model 1 ), where β=log(HR)

**Table S9 to S12 – ONLY WHITE COLLAR WORKERS IN GAZEL**

Table S9 GAZEL WHITE COLLAR WORKERS. Sample characteristics of the British Whitehall II and the French GAZEL cohort studies.

|  | **Occupational position** | | | **Overall** |
| --- | --- | --- | --- | --- |
|  | High | Intermediate | Low |  |
| **WHITEHALL II** |  |  |  |  |
| N (%) | 2914 (29.8) | 4744 (48.6) | 2113 (21.6) | 9771 |
| Deaths (Rate^a^) | 197 (3.1) | 322 (3.8) | 174 (5.2) | 693 (3.6) |
| Mean age (SD) | 45.0 (5.8) | 43.3 (6.0) | 46.0 (6.0) | 44.4 (6.1) |
| **GAZEL** |  |  |  |  |
| N (%) | 1988 (24.6) | 4499 (55.7) | 1592 (19.7) | 8079 |
| Deaths (Rate^a^) | 86 (2.3) | 184 (2.7) | 80 (4.2) | 350 (2.6) |
| Mean age (SD) | 44.1 (3.3) | 42.7 (3.9) | 41.5 (4.1) | 44.3 (3.5) |

SD=Standard Deviation

^a^ Age- and sex-adjusted mortality rate per 1000 person-years

Table S10 GAZEL WHITE COLLAR WORKERS. Association of occupational position with health behaviours in the British Whitehall II cohort (N=9771 at first and N=7166 at last follow-up) and in white-collar workers of the French GAZEL cohort (N=8079 at first and N=6902 at last follow-up).

|  | **WHITEHALL II** | **GAZEL** | P^b^ |
| --- | --- | --- | --- |
|  | **OR^a^ (95%CI)** | **OR^a^ (95%CI)** |  |
| **FIRST FOLLOW-UP** | | | |
| Smoking | 3.68 (3.11, 4.36) | 1.25 (1.05, 1.49) | *<0.001* |
| Heavy drinking | 0.50 (0.42, 0.60) | 0.89 (0.72, 1.09) | *0.24* |
| Unhealthy diet | 7.42 (5.19, 10.60) | 1.32 (1.08, 1.62) | *<0.001* |
| Physically inactive | 6.07 (5.00, 7.36) | 1.99 (1.71, 2.32) | *<0.001* |
| **LAST FOLLOW-UP** | | | |
| Smoking | 4.17 (3.17, 5.47) | 1.12 (0.88, 1.43) | *<0.001* |
| Heavy drinking | 0.36 (0.30, 0.44) | 0.83 (0.67, 1.02) | *0.004* |
| Unhealthy diet | 9.99 (5.66, 17.63) | 2.60 (1.74, 3.88) | *<0.001* |
| Physically inactive | 2.27 (1.92, 2.70) | 1.75 (1.74, 3.88) | *<0.001* |

OR=Odds Ratio; CI=Confidence Interval

^a^ Odds Ratio for lowest versus highest occupational position adjusted for age and sex

^b^ P for interaction between health behaviour and cohort

Table S11 GAZEL WHITE COLLAR WORKERS. The association between health behaviours and all-cause mortality in the British Whitehall II cohort (N=9771, Deaths=693) and in white-collar workers of the French GAZEL cohort (N=8079, Deaths=350).

|  | **WHITEHALL II** | **GAZEL** | P^b^ |
| --- | --- | --- | --- |
|  | **HR** ^a^ **(95% CI)** | **HR** ^a^ **(95% CI)** |  |
| **Smoking** |  |  |  |
| Non smokers | 1.00 | 1.00 |  |
| Current smokers | 2.38 (1.99, 2.85) | 2.21 (1.75, 2.79) | *0.41* |
| **Drinking** |  |  |  |
| Abstainers | 1.56 (1.30, 1.87) | 1.94 (1.49, 2.52) |  |
| Moderate drinkers | 1.00 | 1.00 |  |
| Heavy drinkers | 1.25 (1.02, 1.52) | 1.24 (0.95, 1.63) | *0.73* |
| **Diet** |  |  |  |
| Healthy | 1.00 | 1.00 |  |
| Moderately healthy | 1.41 (1.20, 1.65) | 1.27 (0.97, 1.66) |  |
| Unhealthy | 2.14 (1.49, 3.07) | 2.40 (1.64, 3.52) | *0.61* |
| **Physical activity** |  |  |  |
| Active | 1.00 | 1.00 |  |
| Moderately active | 1.05 (1.49, 3.07) | 1.28 (0.95, 1.73) |  |
| Inactive | 1.60 (1.34, 1.90) | 1.67 (1.31, 2.14) | *0.50* |

HR=Hazard Ratios, CI=Confidence Interval

^a^ Model adjusted for age at baseline and sex

^b^ P for interaction between health behaviour and cohort

Table S12 GAZEL WHITE COLLAR WORKERS. Role of health behaviours used as time dependent covariates in explaining the association between occupational position and all-cause mortality in the British Whitehall II cohort (N=9771, Deaths=693) and in white-collar workers of the French GAZEL cohort (N=8079, Deaths=350).

|  | **WHITEHALL II** | | **GAZEL** | |
| --- | --- | --- | --- | --- |
|  | **HR (95% CI)** | **%Δ ^c^** | **HR (95% CI)** | **%Δ ^c^** |
| Model 1^a^ | 1.62 (1.28, 2.05) |  | 2.26 (1.63, 3.13) |  |
| Model 1 + Smoking | 1.39 (1.09, 1.75) | 32 | 2.20 (1.59, 3.05) | 3 |
| Model 1 + Alcohol | 1.52 (1.19, 1.93) | 14 | 2.14 (1.54, 2.96) | 7 |
| Model 1 + Diet | 1.44 (1.13, 1.83) | 25 | 2.19 (1.58, 3.04) | 4 |
| Model 1 + Physical activity | 1.47 (1.16, 1.86) | 21 | 2.16 (1.56, 3.00) | 5 |
| Fully adjusted Model ^b^ | 1.13 (0.88, 1.44) | 75 | 1.96 (1.41, 2.73) | 17 |

HR=Hazard Ratios, CI=Confidence Interval

^a^ HR for lowest versus highest occupational position adjusted for age at baseline and sex

^b^ HR for lowest versus highest occupational position adjusted for age at baseline, sex, and all health behaviours

^c^ Percent attenuation in log HR= 100 x (β Model 1 - β Model 1+ health behaviour(s))/( β Model 1 ), where β=log(HR)

**Table S13 and S14 – ABSOLUTE PROBABILITIES AND ABSOLUTE DIFFERENCES IN PROBABILITIES**

Table S13 The association between health behaviours and all-cause mortality in the British Whitehall II (N=9 771, Deaths=693) and the French GAZEL (N=17 760, Deaths=908) cohort studies.

|  | **WHITEHALL II** | | | **GAZEL** | | | P^c^ |
| --- | --- | --- | --- | --- | --- | --- | --- |
|  | **Mortality rate ^a^** | **Rate Δ^a^(95% CI)** | **HR** ^b^ **(95% CI)** | **Mortality rate ^a^** | **Rate Δ^a^(95% CI)** | **HR** ^b^ **(95% CI)** |  |
| **Smoking** |  |  |  |  |  |  |  |
| Non smokers | 3.2 | - | 1.00 | 2.7 | - | 1.00 |  |
| Current smokers | 6.2 | 3.0 (0.4, 5.5) | 2.38 (1.99, 2.85) | 5.1 | 2.4 (0.8, 4.0) | 2.11 (1.82, 2.44) | *0.36* |
| **Drinking** |  |  |  |  |  |  |  |
| Abstainers | 4.9 | 1.8 (-0.6, 4.2) | 1.56 (1.99, 2.85) | 4.6 | 1.8 (0.0, 3.6) | 1.89 (1.59, 2.24) |  |
| Moderate drinkers | 3.1 | - | 1.00 | 2.7 | - | 1.00 |  |
| Heavy drinkers | 4.1 | 1.0 (-1.4, 3.4) | 1.25 (1.02, 1.52) | 3.2 | 0.5 (-1.1, 2.1) | 1.14 (0.97, 1.34) | *0.70* |
| **Diet** |  |  |  |  |  |  |  |
| Healthy | 3.4 | - | 1.00 | 2.8 | - | 1.00 |  |
| Moderately healthy | 4.0 | 0.6 (-1.3, 2.6) | 1.41 (1.20, 1.65) | 3.0 | 0.2 (-1.4, 1.7) | 1.17 (0.99, 1.38) |  |
| Unhealthy | 5.8 | 2.4 (-2.8, 7.7) | 2.14 (1.49, 3.07) | 4.4 | 1.6 (-0.9, 4.0) | 2.04 (1.61, 2.60) | *0.49* |
| **Physical activity** |  |  |  |  |  |  |  |
| Active | 3.2 | - | 1.00 | 2.4 | - | 1.00 |  |
| Moderately active | 3.1 | 0.0 (-2.3, 2.2) | 1.05 (0.86, 1.30) | 2.7 | 0.3 (-1.3, 1.9) | 1.22 (1.01, 1.47) |  |
| Inactive | 5.4 | 2.2 (-0.1, 4.5) | 1.60 (1.34, 1.90) | 4.1 | 1.7 (0.2, 3.1) | 1.67 (1.43, 1.95) | *0.45* |

Δ=Difference; HR=Hazard Ratios, CI=Confidence Interval

^a^ Age- and sex- adjusted rate and rate differences calculated using additive models

^b^ Cox regression model adjusted for age at baseline and sex

^c^ P for interaction between health behaviour and cohort, Cox regression

Table S14 Role of health behaviours used as time dependent covariates in explaining the association between occupational position and all-cause mortality in the British Whitehall II (N=9 771, Deaths=693) and the French GAZEL (N=17 760, Deaths=908) cohort studies.

|  | **Rate Δ^c^(95% CI)** | **% attenuation^d^** | **HR^e^ (95% CI)** | **% attenuation^f^ (95%CI)** |
| --- | --- | --- | --- | --- |
| **WHITEHALL II** |  |  |  |  |
| Model 1 (SES, age- and sex- adjusted)^a^ | 1.5 (-1.3, 4.3) |  | 1.62 (1.28, 2.05) |  |
| Model 1 + Smoking | 1.0 (-1.8, 3.9) | 31 | 1.39 (1.09, 1.75) | 32 (20 - 62) |
| Model 1 + Alcohol | 1.2 (-1.6, 4.1) | 18 | 1.52 (1.19, 1.93) | 14 (3 - 37) |
| Model 1 + Diet | 1.3 (-1.6, 4.1) | 16 | 1.44 (1.13, 1.83) | 25 (12 - 55) |
| Model 1 + Physical activity | 1.1 (-1.7, 3.9) | 27 | 1.47 (1.16, 1.86) | 21 (11 - 43) |
| Fully adjusted Model ^b^ | 0.3 (-2.6, 3.3) | 78 | 1.13 (0.88, 1.44) | 75 (44 - 149) |
| **GAZEL** |  |  |  |  |
| Model 1 (SES, age- and sex- adjusted)^a^ | 2.1 (0.1, 4.1) |  | 1.94 (1.58, 2.39) |  |
| Model 1 + Smoking | 2.0 (0.0, 4.0) | 4 | 1.89 (1.51, 2.28) | 4 (2 - 8) |
| Model 1 + Alcohol | 1.9 (-0.1, 4.0) | 6 | 1.85 (1.51, 2.28) | 7 (4 - 11) |
| Model 1 + Diet | 2.0 (0.0, 4.0) | 2 | 1.89 (1.54, 2.33) | 4 (2 - 8) |
| Model 1 + Physical activity | 1.9 (-0.1, 3.9) | 9 | 1.85 (1.50, 2.27) | 8 (4 - 12) |
| Fully adjusted Model ^b^ | 1.7 (-0.3, 3.7) | 19 | 1.71 (1.39, 2.10) | 19 (13 - 29) |

D= Difference; HR=Hazard Ratios; CI=Confidence Interval

^a^ Lowest versus highest occupational position, age- and sex- adjusted

^b^ Lowest versus highest occupational position adjusted for age , sex, and all health behaviours

^c^ Difference in mortality rate per 1000 person-years between lowest and highest occupational position

^d^ Percent attenuation in rate difference=100 x (bModel 1 -bModel 1 + health behaviour(s))/(b Model 1 )

^e^ HR for lowest versus highest occupational position, age- and sex- adjusted

^f^ Percent attenuation in log HR= 100 x (β Model 1 - β Model 1+ health behaviour(s))/( β Model 1 ), where β=log(HR)

^g^ Bias corrected accelerated bootstrap 95% confidence interval for percent attenuation in HR associated to occupational position

**Table S15 and S16 – INVERSE PROBABILITY WEIGHTED**

Table S15 INVERSE PROBABILITY WEIGHTED. Association of occupational position with health behaviours in the British Whitehall II (N=9771 at first and N=7166 &t last follow-up) and the French GAZEL (N=17760 at first and N=15377 at last follow-up) cohort studies.

|  | **WHITEHALL II** | **GAZEL** | P^b^ |
| --- | --- | --- | --- |
|  | **OR^a^ (95% CI)** | **OR^a^ (95% CI)** |  |
| **FIRST FOLLOW-UP** | | | |
| Smoking | 3.67 (3.09, 4.36) | 1.31 (1.17, 1.47) | *<0.001* |
| Heavy drinking | 0.49 (0.41, 0.59) | 1.13 (1.00, 1.27) | *<0.001* |
| Unhealthy diet | 7.42 (5.20, 10.57) | 1.30 (1.14, 1.48) | *<0.001* |
| Physically inactive | 6.10 (4.94, 7.53) | 1.94 (1.75, 2.15) | *<0.001* |
| **LAST FOLLOW-UP** | | | |
| Smoking | 4.16 (3.12, 5.55) | 1.14 (0.97, 1.34) | *<0.001* |
| Heavy drinking | 0.36 (0.29, 0.44) | 0.90 (0.79, 1.02) | *<0.001* |
| Unhealthy diet | 10.12 (5.50, 18.63) | 1.89 (1.46, 2.44) | *<0.001* |
| Physically inactive | 2.29 (1.91, 2.73) | 1.63 (1.47, 1.81) | *<0.001* |

OR=Odds Ratio; CI=Confidence Interval

^a^ Odds ratio for lowest versus highest occupational position adjusted for age and sex

^b^ P for interaction between health behaviour and cohort

Table S16 INVERSE PROBABILITY WEIGHTED. Role of health behaviours used as time dependent covariates in explaining the association between occupational position and all-cause mortality in the British Whitehall II (N=9 771, Deaths=693) and the French GAZEL (N=17 760, Deaths=908) cohort studies.

|  | **WHITEHALL II** | | **GAZEL** | |
| --- | --- | --- | --- | --- |
|  | **HR (95% CI)** | **%Δ ^c^** | **HR** | **%Δ ^c^** |
| Model 1^a^ | 1.68 (1.36, 2.08) |  | 1.98 (1.63, 2.42) |  |
| Model 1 + Smoking | 1.42 (1.12, 1.80) | 32 | 1.90 (1.55, 2.33) | 6 |
| Model 1 + Alcohol | 1.56 (1.22, 2.00) | 15 | 1.86 (1.52, 2.28) | 10 |
| Model 1 + Diet | 1.45 (1.13, 1.85) | 29 | 1.88 (1.53, 2.32) | 8 |
| Model 1 + Physical activity | 1.47 (1.16, 1.88) | 26 | 1.87 (1.52, 2.30) | 9 |
| Fully adjusted Model ^b^ | 1.12 (0.87, 1.45) | 78 | 1.71 (1.38, 2.11) | 22 |

HR=Hazard Ratios, CI=Confidence Interval

^a^ HR for lowest versus highest occupational position adjusted for age at baseline and sex

^b^ HR for lowest versus highest occupational position adjusted for age at baseline, sex, and all health behaviours

^c^ Percent attenuation in log HR= 100 x (β Model 1 - β Model 1+ health behaviour(s))/( β Model 1 ), where β=log(HR)
